# A qualitative approach for a situation analysis of AMR risks in the food animal production sector

**DOI:** 10.3389/fvets.2023.1045276

**Published:** 2023-02-16

**Authors:** Marisa Caipo, María de los Ángeles Gatica, Hernan Rojas, Leopoldo del Barrio

**Affiliations:** ^1^Food and Agriculture Organization of the United Nations, FAO Regional Office for Latin America and the Caribbean, Santiago, Chile; ^2^CERES BCA, Santiago, Chile

**Keywords:** antimicrobial resistance (AMR), qualitative risk assessment, animal production systems, AMR risks in food animals, situation analysis for AMR risks

## Abstract

In response to the need to manage Antimicrobial Resistance (AMR), countries have produced National Action Plans (NAPs), which require detailed information of the AMR situation in all sectors. Considering the limited information that is publicly available for an analysis of the AMR situation in animal production, the FAO Regional Office for Latin America and the Caribbean (FAO RLC) developed the “FAO tool for a situation analysis of AMR risks in the food and agriculture sectors.” The objective of this paper is to present the methodology developed for a qualitative evaluation of the risk factors of AMR toward animal and human health, based on terrestrial and aquatic production systems and their associated national public and private mitigation measures. The tool was developed reflecting the AMR epidemiological model and the guidelines to conduct a risk analysis of AMR from the *Codex Alimentarius* and WOAH. Applied in four stages of progressive development, the objective of the tool is to provide a qualitative and systematic assessment of the risks of AMR from animal production systems, to animal and human health, and to identify gaps in cross cutting factors in AMR management. The tool consists of three instruments: (i) a survey to collect data for a situation analysis of AMR risks; (ii) a methodological procedure for the analysis of the information obtained; (iii) instructions for the preparation of a national roadmap for the containment of AMR at a national level. Based on the results from the information analysis, a roadmap is prepared by guiding and prioritizing the needs and sectoral actions for the containment of AMR under an intersectoral, multidisciplinary and collaborative approach, and according to country priorities and resources. The tool helps to determine, visualize and prioritize the risk factors and challenges that contribute to AMR from the animal production sector and that need to be addressed to manage AMR.

## 1. Introduction

Antimicrobials are critical elements in the food and agriculture sector, contributing to sustainable terrestrial and aquatic animal production systems. Although Antimicrobial Resistance (AMR) is known to be part of a natural selection for bacterial agents (1, 2), the use, abuse and misuse of antimicrobials have contributed to the development and spread of AMR (3).

AMR has prompted a global health crisis which knows no geographical, political or economic barriers. Scientific evidence shows that with the emergence of bacteria now resistant to substances to which they were once susceptible, infections are increasingly difficult to treat. Thus, AMR constitutes a serious threat to human and animal health. From an economic standpoint, the World Bank reports that by 2050 AMR could cause a decrease in the world's gross domestic product (GDP) of 1.1–3.8 percent per annum. During the same timeframe, health costs could also increase between USD 0.33 trillion and over USD 1 trillion per year. Livestock is especially exposed to AMR impacts, by 2050 a loss of 11 percent of livestock production is expected in low-income countries (4). In the food production sector, AMR puts food safety, food security and economic wellbeing of millions of households at risk (5). Animal health is also affected by AMR, with a decrease in the effectiveness of antimicrobial treatments (6).

Recognizing the importance and impact of AMR, and in response to the Global Action Plan for AMR (7), countries have produced National Action Plans (NAPs) that outline strategies to combat and manage AMR from all sectors, including components related to food and agriculture.

One of the first steps for the implementation of a NAP demands that responsible health authorities have information detailing the AMR situation, thus enabling them to design, implement and manage appropriate strategies for each country (8).

The use of antimicrobials in animal production, human health and agriculture is one of the most important elements in the generation of AMR. The epidemiological interdependence between humans, animals and the environment highlights the importance of addressing the risk factors that contribute to the introduction and exposure of AMR from animal production (6).

The exploration of the risk pathways associated with AMR in the food chain in the Latin American and Caribbean region revealed a significant lack of information that would enable a systematic quantitative/semi-quantitative assessment (9). One approximation to this challenge is the First Annual Report on the Use of Antimicrobial Agents in Animals of the World Organization for Animal Health (WOAH) (10). A second approach is the Tripartite AMR country self-assessment survey (TrACSS), focused on monitoring the country progress in the implementation of the national actions plans. The first results of this multi-sectoral self-assessment surveys were delivered in 2016, with little information from the food and agriculture sector.

Risk analysis is a process that can assess the health risks of AMR as well as identify and define the necessary strategies to manage and reduce these risks (11). Risk assessment is a systematic process that, with the knowledge available, intends to understand the nature of the risk involved, express and evaluate that risk (12). Risk assessment, as defined by the *Codex Alimentarius*, is a scientifically based process consisting of the following steps: (i) hazard identification; (ii) hazard characterization; (iii) exposure assessment; and (iv) risk characterization (13).

Due to the complex nature of AMR, an expert group appointed by the WOAH developed a risk analysis process which considered different approaches to risk assessment (14), with the understanding that risk assessment principles apply to both quantitative and qualitative risk assessments (15, 16). Qualitative risk assessments have been conducted to answer questions related to animal health, particularly where scarce data exists (17). The inclusion of expert opinion and ease of understanding for all stakeholders are positive characteristics of this type of assessment. Qualitative risk assessment has assisted risk managers in mitigating risks and communicating decisions (17).

Due to the limited information required that is publicly available for an analysis of the AMR situation in animal production, in 2017, the FAO Regional Office for Latin America and the Caribbean (FAO RLC) developed the “FAO tool for a situation analysis of AMR risks in the food and agriculture sectors” (hereafter “FAO situation analysis tool”). This tool was developed under the One Health approach, essential for the containment of AMR, to provide a qualitative and organized assessment of AMR risks from animal production systems.

Given that the countries did not have specific AMR data (epidemiology/microbiology) for the food and agriculture sector at that time, this tool aims to be a first approach on the general aspects of AMR, taking into account the risks and the mitigation measures (regulation, private or public standards and programs related to good practices) without considering any specific characteristics of a particular agent.

The objective of this paper is to present the methodology developed by FAO for a qualitative evaluation of the risks of AMR to animal and human health (18). The evaluation is based on terrestrial and aquatic production systems and their associated national public and private mitigation measures.

This tool was initially developed with antibiotics as the main antimicrobial of concern in animal production. It allows the evaluation of all types of antimicrobials, but prior to its application, questions should be revised to reflect the specific antimicrobial to be assessed.

## 2. Methodology

**Figure 1:**
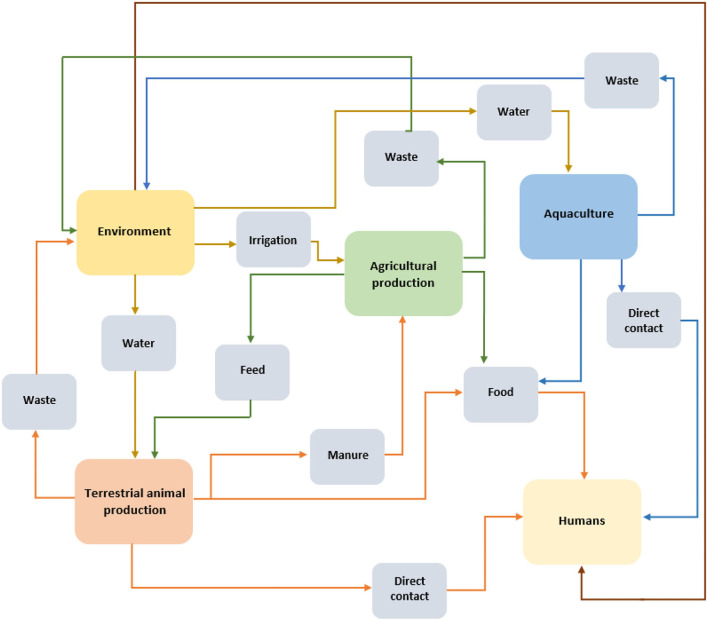
A simplified representation of AMR epidemiology routes from antimicrobial use in animal production.

The FAO RLC team developed the FAO situation analysis tool to assess the risks of AMR from animal production toward animal health and human health. The tool was generated considering the AMR epidemiological model (Figure 1), and the guidelines for conducting a risk analysis for AMR from the *Codex Alimentarius* (19) and WOAH (15, 16). This approach identifies the data required for a risk assessment, considering the AMR risk factors, the respective mitigation measures for AMR control, and cross cutting elements that influence AMR.

The development of the tool was comprised of three steps: (i) identifying information related to AMR in the animal production chain; (ii) designing the information collection and analysis process; and (iii) validating the tool.

### 2.1. Identification of information

The FAO RLC team assembled an exhaustive review of literature from international organizations, country studies and scientific papers related to AMR and AMU in food animal production systems. With this information, FAO RLC team identified the epidemiological routes of AMR from the use in animal production to animal health, human health and the environment. Identifying the epidemiological routes of AMR contributes to the design, application and evaluation of successful mitigation measures (6).

The generation and dissemination of any infectious agent in an animal production system depends on multiple factors such as the conditions in which the animals are kept, feeding, biosecurity measures, and the use of preventive elements such as vaccination, among others. Despite the measures applied, the risk of existence of these agents persists. Therefore, resources such as antimicrobials are used to tackle or prevent the diseases caused by infectious agents, practices that influence the generation of AMR. The risk of AMR spread from animal production to the animals themselves is related to direct contact between the animals, waste disposal and the contamination of the environment (including water sources). For humans, these exposure routes are depicted as transmission through environmental pollution, contaminated food, and direct contact with animals and products from the productive system (6).

However, the generation and dissemination of AMR is also influenced by other cross cutting factors. These include regulatory policies, the effectiveness of sanitary programmes associated with antimicrobial use (AMU) in animal production, and the existence and exchange of information regarding AMR in animal and human health. Equally important are the data for the implementation and monitoring of strategies, the use of alternatives to antimicrobials, the collaboration mechanisms under One Health, and research (20).

### 2.2. Design of the information collection and analysis process

The necessary data was identified from the literature review. The data to be collected, included factors from the AMR epidemiological model (Figure 1), public and private measures that mitigate AMR risks, and other cross cutting elements. The latter were based on the priority areas of the FAO Action Plan on AMR 2016–2020 (21). As the foundation of the system, the cross-cutting elements clearly influence the generation and spread of AMR.

Figure 1 is a schematic for the pathways of AMR dissemination to and from animal production and the relationship with the environment, agricultural production and humans. The arrows represent the direction of possible AMR flows from an intersectoral view.

A survey was designed to gather information and initiate and/or strengthen intersectoral work by the participation of national technical teams from both the public and private sectors. The survey is led by the Animal Health Authority (as FAO counterpart) for the collection of information. The survey comes with a list of instructions, including the list of stakeholders that should participate during the collection of information (Annex 1).

The information analysis was based on risk assessment principles from the *Codex Alimentarius* (19) and WOAH (15, 16). A procedure was established to build a national roadmap that, based on the results from the information analysis, would identify feasible solutions for the management of AMR risks or gaps in the country.

### 2.3. Tool validation

The content and design of the survey questions were validated, as was the analysis method for the information to be gathered. Five workshops were conducted to apply expert elicitation processes on the components of the tool, where international experts from the public, private and academic sectors from Latin America participated in this process. The experts contributed with their experience in the areas of terrestrial and aquatic animal production systems, family farming, public health and risk analysis.

The expert elicitation processes consisted of a detailed assessment of each question and possible answers. The experts held a joint discussion on each survey component of the tool to reach consensus on the final text and improve the information to be collected, the survey questions and method of analysis.

The tool was constructed in Spanish and English, reflecting the multilingual environment of Latin America and the Caribbean, and allowing for its application in other regions of the world. During the year 2020–2021 the tool was piloted in two African countries. If necessary, the tool can be easily adapted to the national conditions where it will be applied.

## 3. Results

The FAO situation analysis tool was developed with the objective to provide a qualitative and systematic national assessment of the risks of AMR from animal production systems (terrestrial and aquatic species) to animal and human health.

**Figure 2:**
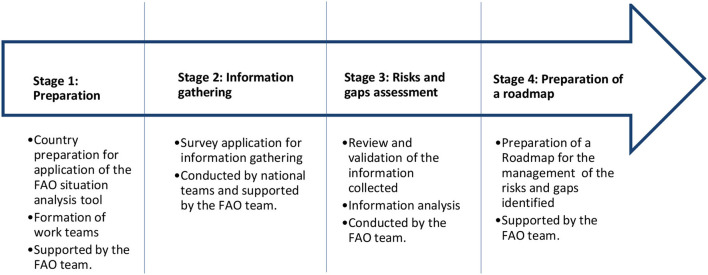
Stages for the application of the FAO situation analysis tool.

The tool consists of three main instruments applied in four stages of progressive development with the support of the FAO team (Figure 2):

- A survey for data collection to generate a situation analysis of AMR risks, including instructions for its application, indicating the stakeholders considered for each of the components;
- A methodological procedure for the analysis of the information obtained through the survey, and
- Instructions for the development of a national roadmap for the containment of AMR.

In general, Stage 1 is allotted a period of 2 weeks. Stage 2 takes 2 months on average. Stage 3 is finalized in 1 month.

### 3.1. Survey for data collection to generate a situation analysis of AMR risks

The purpose of the survey is to obtain information to evaluate: (i) risk factors toward animal and human health (understood as the epidemiological routes of AMR) related to the introduction and exposure of AMR from animal production; (ii) existence and effectiveness of public/private programs and regulatory elements, as well as private mitigation measures, which contribute to the control of AMR risk factors; and (iii) gaps in cross cutting factors that enable system sustainability.

The survey has 322 questions organized into four components (the list of the questions is presented in Annex 2):

- Component 1: Terrestrial animals;
- Component 2: Aquatic animals;
- Component 3: Family farming; and
- Component 4: System sustainability factors.

Components 1, 2, and 3 include questions related to the risk factors (Tables 1–3) and subfactors that correspond to the epidemiological pathways that generate and disseminate AMR (Figure 1). For components 1 and 2, the information is collected from seven animal production systems: broiler chickens, laying hens, pigs, beef cattle, milk cattle, fish, and crustaceans. Family farming is a productive system that usually produces more than one species within the same system. Hence, the information is gathered as terrestrial and aquatic species (with the opportunity to deliver the information for a particular species). Component 4 incorporates questions about the cross cutting factors related to AMR (surveillance, governance, communication, awareness and research).

**Table 1:** AMR introduction risk factors and subfactors for animal and human health.

**Risk factor**	**Risk subfactor**
Characterization of the production system	- Animal population
Sanitary conditions in animal production	- Prevalent infectious agents/pathologies treated with antibiotics
Farming practices in animal production	- Animal population records
	- Animal identification system
	- Animal identification system that allows traceability
	- Disease records
	- Morbidity records
	- Mortality records
	- Animal management records
	- Veterinary assistance or other professional recognized by the competent authority
	- Diagnosis carried out by a professional or legally qualified technician
	- Application of measures for internal biosecurity
	- Application of measures for external biosecurity
	- Application of animal welfare measures
Practices of antibiotics use in food producing animals	- Classification of antibiotics used within the WOAH List of Antimicrobial Agents of Veterinary Importance (22) and WHO List of Critically Important Antimicrobials for Human Medicine (23)
	- Use of authorized selling points for antibiotics
	- Production system is dependent on the use of antibiotics
	- Decision on the use of antibiotics made by a legally qualified person or veterinarian
	- Administration of antibiotics supported by a prescription from a legally qualified person or veterinarian
	- Prescription of antibiotics is accompanied by instructions for their use
	- Recommendations made by the manufacturer are followed
	- Use of antibiotics for therapeutic and preventive purposes (prophylaxis/metaphylaxis) and growth promotion
	- The person administering antibiotics is trained
	- Use of antibiotics that are authorized and registered by competent authority
	- Registry for the administration of antimicrobials
	- Observance of withdrawal periods
	- Storage of antibiotics follows manufacturer recommendations
	- Administration of antibiotics respecting their expiration date
Feed practices	- Mislabelling of feed containing antibiotics
	- Processing of feed follows procedures to control physical and microbiological contamination
	- Feed is produced in separate production lines for medicated and non-medicated
	- Feed transported and distributed following procedures to control physical and microbiological contamination
	- Feed is manufactured with traceability processes
	- Existence of a traceability system for medicated concentrated feed from production to the farm of destination
	- Application of measures to reduce physical and microbiological contamination in the management and storage of feed
	- Auditable registry of feed used in animal production
	- Use of drinking water for animal consumption
	- The decision on use of antibiotics is made by legally qualified person or veterinarian
	- Manufacturing of medicated feed with antibiotics is supported by a legally qualified person or veterinarian
	- Prescription of medicated feed with antibiotics is accompanied by instructions of application
	- Follow-up of the manufacturer's recommendations in the application of antibiotics in feed
	- Use of medicated feed with antibiotics for therapeutic, preventive or growth promotion purposes
	- The person in charge of delivering feed is trained
	- Antibiotics used are authorized and registered by the competent authority
	- Maintenance of a medicated feed application registry
	- Observance of withdrawal period for medicated feed
	- Medicated feed is stored and preserved in appropriate conditions during transport
	- Medicated feed is stored and preserved in appropriate conditions.

**Table 2:** AMR exposure risk factors and subfactors for animal health.

**Risk factor**	**Risk subfactor**
Sanitary conditions in animal production	- Prevalent infectious agents / pathologies treated with antibiotics
Farming practices in animal production	- Animal population records
	- Animal identification system
	- Animal identification system that allows traceability
	- Disease records
	- Morbidity records
	- Mortality records
	- Animal management records
	- Veterinary assistance or other professional recognized by the competent authority
	- Diagnosis carried out by a professional or legally qualified technician
	- Application of measures for internal biosecurity
	- Application of measures for external biosecurity
	- Application of animal welfare measures
Environmental management practices	- Methods used to dispose of guano in farms
	- Methods used to dispose of slurry/manure in farms
	- Methods used to dispose of dead animals in farms
	- Methods used to dispose of medicated feed in farms
	- Methods used to dispose of antibiotics in farms
	- Methods used to dispose of containers of antibiotics in farms
	- Methods used to dispose of medicated feed in animal feed production stations
	- Methods used to dispose of antibiotics in animal feed production stations
	- Methods used to dispose of containers of antibiotics in animal feed production stations
	- Methods used to dispose of dead animals in slaughterhouses
	- Methods used to dispose of animal waste and products seized at slaughterhouses.

**Table 3:** AMR exposure risk factors and subfactors for human health.

**Risk factor**	**Risk subfactor**
Consumption of food of animal origin	- Consumption per capita
	- Consumption of raw products
Consumption of food of animal origin contaminated with bacteria	- Existence of food contaminated with bacterial agents (surveillance programme)
Consumption of food of animal origin contaminated with antimicrobial residues	- Existence of food contaminated with antimicrobial residues (surveillance programme)
Direct contact with food-producing animals and animal products	- Number of people at national level employed in animal production and animal products processing plants
Environmental management practices	- Methods used to dispose of guano in farms
	- Methods used to dispose of slurry/manure in farms
	- Methods used to dispose of dead animals in farms
	- Methods used to dispose of medicated feed in farms
	- Methods used to dispose of antibiotics in farms
	- Methods used to dispose of containers of antibiotics in farms
	- Methods used to dispose of medicated animal feed in animal feed production stations
	- Methods used to dispose of antibiotics in animal feed production stations
	- Methods used to dispose of containers of antibiotics in animal feed production stations
	- Methods used to dispose of dead animals in slaughterhouses
	- Methods used to dispose of animal waste and products seized at slaughterhouses.

The application of the survey is led by the Animal Health Authority, with support of a country team and following guidance provided by the FAO team. The instrument is also accompanied by written recommendations of the stakeholders that should be included in the multisectoral, multidisciplinary and collaborative technical teams, as well as methods for collecting information (survey recommendations in Annex 1).

### 3.2. Methodological procedure for the analysis of the information obtained through the survey

The methodology for the analysis of information is divided into two parts: (i) the qualitative assessment of AMR risks from animal production and their effects on animal and human health; and (ii) an evaluation of gaps of the system sustainability factors.

#### 3.2.1. Qualitative risk assessment of AMR risks

**Figure 3:**
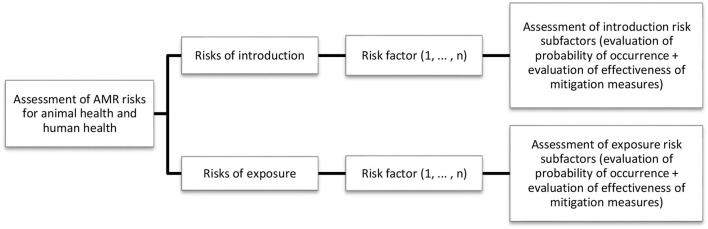
General process for the qualitative assessment of AMR risks in animal and human health.

This assessment is based on the hazard, risks of introduction, risks of exposure, and mitigation measures associated with each of the introduction and exposure risks (Figure 3)^1^. The risks of introduction and exposure are structured into risk factors and subfactors (Tables 1–3), as they relate to the corresponding epidemiological pathways. The mitigation measures incorporated (and analyzed according to each risk subfactor) correspond to public and private measures (regulatory elements, programs) in force in each country (for details see Annex 3). A review of animal health risk assessments was carried out to investigate the relationship between risk factors and mitigation measures, with only one result for African swine fever (17). To the best of the authors' knowledge, no previous risk assessments have considered the relationship between AMR risk factors and existing mitigation measures. A study developed in Southeast Asia proposes a similar method the same year the FAO situation analysis tool was designed (24).

Each risk subfactor and mitigation measure is evaluated as per criteria developed by the FAO team and validated by international experts from Latin America through an expert elicitation process (Tables 4, 5). The final risk of each subfactor is obtained by means of a qualitative risk assessment matrix, which relates the probability of occurrence to the effectiveness of the associated mitigation measures (Table 6). An example of a qualitative risk assessment for the productive practices factor is shown in Table 7.

**Table 4:** Criteria to estimate risk occurrence probability.

**Probability of risk occurrence**	**Definition**
High	Given the characteristics of the hazard and the situation analyzed, the event occurs frequently (always or almost always).
Moderate	Given the characteristics of the hazard and the situation analyzed, the event occurs or may occur on a regular basis (on some occasions).
Low	Given the characteristics of the hazard and the situation analyzed, the event is rare, but does occur.

**Table 5:** Criteria to estimate effectiveness of mitigation measures.

**Effectiveness of mitigation measures**	**Definition**
High	Mitigation measures are effective in preventing or controlling the hazard.
Moderate	Mitigation measures are moderately effective in preventing or controlling the hazard.
Low	Mitigation measures are not very effective in preventing or controlling the hazard.

**Table 6:** Risk estimation matrix.

**Probability of occurrence**	**Effectiveness of mitigation measures**		
	**High**	**Moderate**	**Low**
High	Low	Moderate	High
Moderate	Low	Moderate	Moderate
Low	Low	Low	Low

**Table 7:** Example^*^ of a qualitative risk assessment for farming practices in animal production.

**Factor: farming practices in animal production**			
**Subfactor**	**Estimation of the probability of risk subfactor occurrence**	**Estimation of the effectiveness of mitigation measures**	**Final risk estimation**
Animal population records	Moderate	Moderate	Moderate
Animal identification system	Moderate	High	Low
Animal identification system that allows traceability	Low	High	Low
Disease records	High	High	Low
Morbidity records	Moderate	High	Low
Mortality records	Moderate	High	Low
Animal management records	Moderate	High	Low
Veterinary assistance or other professional recognized by the competent authority	Low	High	Low
Diagnosis carried out by a professional or legally qualified technician	Low	Moderate	Low
Application of measures for internal biosecurity	Moderate	Moderate	Moderate
Application of measures for external biosecurity	Moderate	Moderate	Moderate
Application of animal welfare measures	Low	Moderate	Low

* This example does not represent any particular species.

The risk assessment process is carried out independently for animal and human health, and for each animal production system evaluated, distinguishing between intensive production and family farming.

#### 3.2.2. Identification of gaps of the system sustainability factors

The analysis of sustainability factors consists of identifying gaps in the system using the criteria developed by the FAO team and validated by international experts from Latin America through an expert elicitation process. The factors analyzed are Surveillance of the use of antibiotics, AMR surveillance, Institutional governance of One Health and the agri-food sector, Communication, awareness and training, and Research and innovation. These are comprised of 7, 18, 36, 23, and 2 subfactors, respectively (Annex 3, Component 4: System sustainability factors).

The example presented in Table 7 is a direct representation of how the countries perceive the risks (of risk factors and subfactors) identified in each of the animal production systems analyzed.

A more detailed example to facilitate the understanding of the methodology applied for different risk factors in broiler chickens, is presented in Annex 3. This example does not represent any specific country situation and is not based on actual data.

### 3.3. Instructions for the development of a national roadmap for the containment of AMR

The roadmap is prepared through a national workshop. By adopting an intersectoral, multidisciplinary and collaborative approach, the workshop incorporates stakeholders from the official veterinary and food safety services, along with other representatives of the terrestrial and aquatic animal production sector (public and private), environment and the human health sector. These include technical staff, decision makers and policymakers on AMR.

Based on the analysis of the information collected from the survey, the roadmap is prepared by guiding and prioritizing national needs and sectoral actions for the containment of AMR. Actions are outlined according to the characteristics of the productive, health and institutional systems of each country, and in line with the National Action Plan on AMR, the Global Action Plan on Antimicrobial Resistance (7), and the FAO Action Plan on Antimicrobial Resistance (21, 25).

The roadmap guides decision-making based on evidence and mitigation measures according to country technical, legal and economic feasibility (the tool includes instructions for roadmap application to facilitate the process). Consequently, the country has an agreed instrument for the construction, strengthening and monitoring of the national strategy for the containment of AMR in the food and agriculture sector.

The steps to formulating a roadmap are:

- Step 1. Awareness and prioritization of risks and gaps. Country stakeholders prioritize the risks and gaps recognized through the qualitative risk assessment and gap identification. The prioritization of risks should be carried out together with other relevant actors from the public and/or private sectors.
- Step 2. Identification of solutions for prioritized risks and gaps. For each solution, the internal or external factors (political, administrative, economic, or operational) are defined, along with the actors that could facilitate or restrict the application and/or effectiveness of the proposed solutions.
- Step 3. Analysis and proposal for the management of stakeholders. This step consists of the identification and characterization of the interest groups related to the containment of AMR (from the public or private sector, national or international). The role of each stakeholder within the roadmap is also identified.
- Step 4. Development of the roadmap. The identified actions or solutions are ranked. Each action or solution is characterized in relation to its verifiable product, the person or unit responsible, start and end dates, the resources involved, and other necessary requirements.

## 4. Discussion

Many international agencies have developed guides, databases and tools to support countries in the management of AMR (26–31). However, to the best of the authors' knowledge, the FAO situation analysis tool is the first to provide countries with an evaluation of the AMR risks and gaps of the entire animal production chain, considering all actors involved. Through consideration of epidemiological pathways, their associated mitigation measures, and the sustainability elements of the system, the FAO situation analysis tool incorporates most aspects relating to the generation and spread of AMR. The results also provide a picture of the current situation and guide decision-making for the containment of AMR under the One Health approach, in line with country needs and resources.

The constant support of the FAO team during the application of the tool facilitates its implementation across different settings, addressing the challenges related to the collection of information. To date, the FAO situation analysis tool has been applied in ten countries in Latin America and the Caribbean, and two countries in Africa. The results show the feasibility of carrying out a qualitative assessment of the risks of AMR from food animal production to animal and human health.

The FAO situation analysis tool is designed to be applied in any country, regardless of its economic classification, thereby offsetting the centralization of information from high-income countries (32).

Risk assessment is often regarded as a complex process due to the amount of information it requires. The application through this tool in two geographical regions shows the existence of information (whether aggregated or not) and the capacity to obtain relevant information in the countries to develop a risk assessment.

The methodology has a global and highly specific approach to AMR risk management. From the global point of view, the risk factors applicable to risk assessment and associated mitigation measures are identified (both regulatory and others, from the public and private sectors), along with fundamental aspects of the system's sustainability. At the same time, the specificity of the tool enables the evaluation of those risk factors with the associated mitigation measures, and sustainability elements that are generally not characterized, both challenges are rendered invisible in the daily tasks of animal production. Examples of this include the insufficient use of records in the application of good production practices, the lack of regulations developed which pertain to the use of antibiotics in food for animal consumption and environmental management practices. Regarding the evaluation of the mitigation measures (regulatory or public/private programs), the specificity of the tool allows the country not only to evaluate the existence of the regulation, but also the effectiveness of the application of this measure (Annex 3).

The structure of the FAO situation analysis tool allows the country to look at the epidemiological and system sustainability factors that influence AMR and highlights the importance of taking a systematic approach to address One Health challenges.

The application of the FAO situation analysis tool enables the identification of the weakest factors to address the containment of AMR. Tables 8, 9 show, for each risk factor and animal production system, challenges identified (as a percentage) through the qualitative risk assessment for six participating countries of Latin America and the Caribbean. The results revealed inadequacies in the sanitary condition of animals, antimicrobial use practices and environmental management practices, respectively (see shaded results in Table 8). In relation to human health, the consumption of food of animal origin, the consumption of food of animal origin contaminated with bacteria, and the direct contact with animals or animal products (see shaded results in Table 9) were identified as the factors with higher risks (18).

**Table 8:** Percentage of subfactors that present risks^*^ based on the qualitative risk assessment for animal health, by risk factor and animal production system.

Risk factor (^**^)	**Broiler chickens**	**Laying hens**	**Pigs**	**Beef cattle**	**Milk cattle**	**Fish**	**Crustaceans**
**Introduction risk**							
Sanitary conditions (3)	100	100	100	67	33	0	100
Farming practices (12)	25	25	42	25	25	8	33
Antimicrobial use practices (14)	57	50	64	43	29	36	71
Feed practices (20)	45	40	45	15	10	0	10
Total introduction risk (49)	47	43	53	29	20	12	39
**Exposure risk**							
Sanitary conditions (animal health) (3)	100	100	100	67	33	0	100
Farming practices (12)	25	25	42	25	25	8	33
Environmental management practices (11)	55	55	64	36	45	36	73
Total exposure risk (26)	46	46	58	35	35	19	58
Total introduction + exposure risk (75)	47	44	55	31	25	15	45

* A risk is identified when at least 50 percent of the participating countries present a high or moderate final risk in the evaluated subfactor.

** Total number of subfactors within each risk factor (as seen in Tables 1, 2).

The shaded cells seek to highlight when 50% of the risk sub-factors evaluated within each risk factor present a moderate to high level of risk among the countries assessed.

**Table 9:** Percentage of subfactors that present risks^*^ based on the qualitative risk assessment for human health, by risk factor and animal production system.

Risk factor (^**^)	**Broiler chickens**	**Laying hens**	**Pigs**	**Beef cattle**	**Milk cattle**	**Fish**	**Crustaceans**
**Introduction risk**							
Sanitary conditions (3)	100	0	33	67	0	33	67
Farming practices (12)	25	25	42	25	25	8	33
Antimicrobial use practices (14)	57	50	64	43	29	36	71
Feed practices (20)	45	40	45	15	10	0	10
Total introduction risk (49) Exposure risk	47	37	49	29	18	14	37
Environmental management practices (11)	55	55	64	36	45	36	73
Consumption of food of animal origin (2)	50	50	0	0	50	50	100
Consumption of food of animal origin contaminated with bacteria (1)	100	0	100	100	100	100	0
Consumption of food of animal origin contaminated with antimicrobial residues (1)	100	0	0	0	0	100	0
Direct contact with food-producing animals and animal products (1)	100	0	100	100	100	0	0
Total exposure risk (16)	63	44	56	38	50	44	63
Total introduction + exposure risk (65)	51	38	51	31	26	22	43

* A risk is identified when at least 50 percent of the participating countries present a high or moderate final risk in the evaluated subfactor.

** Total number of subfactors within each risk factor (as seen in Tables 1, 3).

The shaded cells seek to highlight when 50% of the risk sub-factors evaluated within each risk factor present a moderate to high level of risk among the countries assessed.

In some cases, such as in the consumption of food of animal origin, the consumption of food of animal origin contaminated with bacteria, and the direct contact with animals or animal products, the high percentage of risks may be caused by the lack of data in the countries (Tables 8, 9). One assumption made by the methodology is that the lack of data is evaluated with a high probability of occurrence; depending on the mitigation measures in each country, this can lead to either high or moderate final risks. Future evaluations may show changes in the results, to the extent that countries develop or strengthen their surveillance or data collection systems.

Related to system sustainability factors, important gaps were observed in the surveillance of the use of antibiotics, AMR surveillance and risk communication.

**Figure 4:**
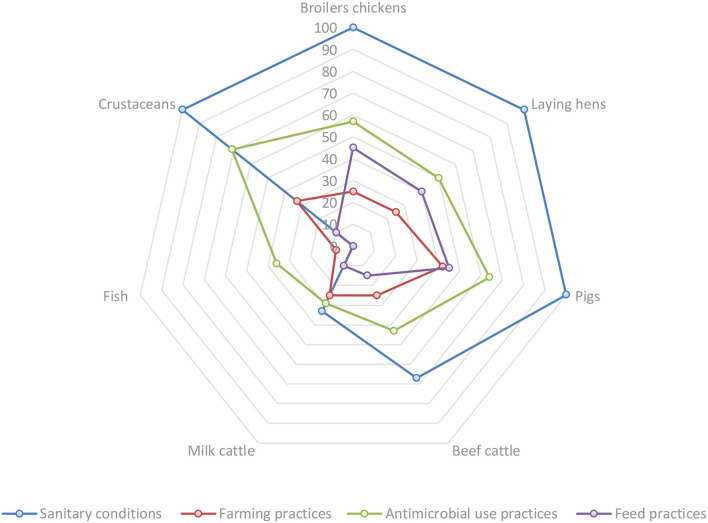
Comparative percentages of subfactors that present risks based on the qualitative risk assessment for animal health, by introduction risk factors and animal production systems.

**Figure 5:**
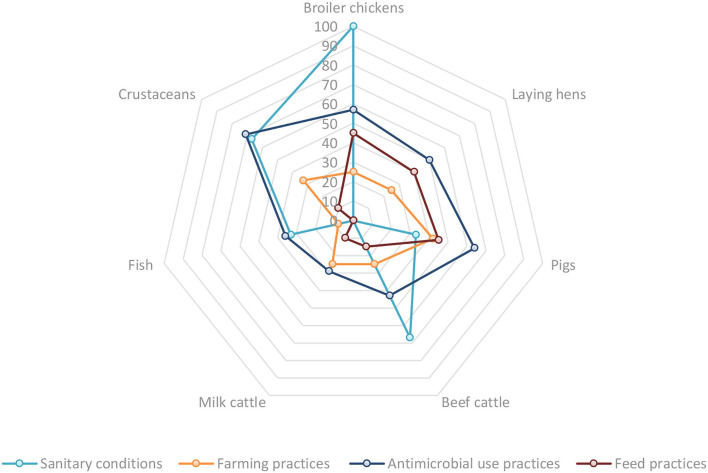
Comparative percentages of subfactors that present risks based on the qualitative risk assessment for human health, by introduction risk factor and animal production systems.

The FAO situation analysis tool shows the differences between animal production systems in the countries. For example, broiler chickens, laying hens, pigs and crustaceans show higher risks (Tables 8, 9). Figures 4, 5 show a comparative view between each animal production system by introduction and exposure risk factors toward animal and human health, respectively.

Figure 4 illustrates the importance of sanitary conditions as a risk factor for the introduction of AMR toward animal health in crustaceans, pigs, broilers and laying hens. Another relevant risk factor to animal health is found in Antimicrobial risk practices, especially for crustaceans, pigs and broiler chickens.

Figure 5 illustrates the relevance of sanitary conditions from broilers, beef cattle and crustaceans to human health.

The consumption of food of animal origin contaminated with bacteria, and direct contact with animals and animal products are significant exposure risk factors for human health, especially in broiler chickens, pigs and cattle.

The identification of the weakest factors and the differences between animal production systems allow countries to prioritize their risks and gaps, facilitating the formulation of a roadmap to address the containment of AMR.

The independent evaluation of intensive production and family farming allows to identify specific risks for each of these productive systems, and highlights the importance of the participation of all productive sectors in AMR management. The need for specific characterization and the availability of differential policies, programs and/or regulations developed and implemented in an effective way for this sector is evident in family farming.

The FAO situation analysis tool is designed to be applied to seven animal production systems. The country has the possibility of applying the tool in some or all seven, or it can propose new animal production systems. Moreover, the independent evaluation between intensive animal production systems and family farming also allows the country to opt for evaluation in both areas.

The tool has shown that the containment of AMR not only requires specific measures but can also be managed indirectly. The latter includes adequate sanitary management; the application of good animal production practices; the implementation of biosecurity and animal welfare measures; good practices in the production and handling of food for animal consumption; adequate hygiene practices during the processing of foods of animal origin, among others.

The journey from information gathering to roadmap formulation contributes to participatory instances between the different actors involved in the animal production chain. This enhances the understanding and awareness of the risks derived from AMR, increases the participatory and collaborative intersectoral work in the public and private sectors, and emphasizes shared responsibility in containing AMR. At the same time, it also increases the participation of the agri-food sector in the National Intersectoral Committee on AMR.

Countries address multiple health topics in the agri-food sector simultaneously. The FAO situation analysis tool facilitates the establishment of priorities for the AMR challenges identified within the animal production sector, and allows high-level decision makers to visualize those challenges, enhancing the importance of AMR within the political agenda of the food sector. On the other hand, the results reveal country strengths regarding the construction of general and specific policies and strategies for the containment of AMR.

As AMR is a topic of global concern, the development of public policies for AMR management could be necessary for some countries, and the adequate technical, legal, economic, and political inputs are fundamental elements for policy success. One outcome of the tool's application was a document that provides technical and legal elements for the construction and implementation of a national public policy for AMR, with an emphasis on animal production (33). This document also provides arguments to corroborate the importance of addressing AMR within the country's strategic agenda for use by the veterinary and food safety services.

## 5. Limitations

Despite the results obtained, the tool is subject to constant evaluation and refinement.

The tool is particularly dependent on intersectoral participation to reflect the animal production chain and the impact of AMR risks on animal and human health. Although the tool seeks to be implemented under an intersectoral approach and suggests its importance, it was not created with the evaluation of that approach in mind. The challenge of addressing AMR from the One Health approach, requiring strong commitment and intersectoral participation, is a recent effort that may suffer delays due to the organizational structure of the National Official Services and the relationship between public institutions and with the private sector, respectively. However, the gradual strengthening of the One Health approach should facilitate tool application.

This version of the tool requires the support of the FAO team in each stage of its application. In the future, the tool will undergo sufficient refinement to be applied in the self-assessment format. This will give countries the autonomy to undertake its periodical application without FAO intervention.

Some of the information requested may exist in a disaggregated form, be difficult to access or not exist at all, skewing the results toward greater risks. However, the results from the tool's application in the country represent the current situation, which may change over time. Final risk results may be greater or lesser, depending on the data available (Table 7).

For the purposes of the tool, the methodology for the information analysis was developed under a regional context. However, the evaluation criteria could be adjusted according to the specific needs of each country with the input of national experts.

Finally, as with any tool, the results will depend on the quality of the data provided.

## 6. Conclusions

The FAO situation analysis tool helps to determine and visualize the risk factors involved in the process of AMR introduction and exposure from animal production. The tool allows the identification and collection of information from the different sectors of animal production. The analysis highlights, lists and prioritizes the challenges to be addressed for the management of AMR at the technical level, but also at the level of authorities and decision makers, based on country priorities and resources. In the public sector, it strengthens the relationship between the areas or institutions of national services, together with the private sector, academia, and other actors. For the private sector, the tool demonstrates the importance of the problem and reinforces sectoral participation in the search for joint solutions with the public sector under the One Health approach.

## Data availability statement

The original contributions presented in the study are included in the article/Supplementary material, further inquiries can be directed to the corresponding authors.

## Author contributions

All authors listed have made a substantial, direct, and intellectual contribution to the work and approved it for publication.

## Footnotes

^1^A “hazard” is a microorganism that has acquired resistance—or resistance genes – as a result of the use of antimicrobials in animal production. “Risks of introduction” refer to the pathways for the appearance of AMR in bacterial populations or the transmission of resistance genes between bacteria, as a result of the use of antimicrobials in animal production. “Exposure risks” are pathways of exposure to AMR bacteria or resistance genes for humans and animals. “Mitigation measures” are all those measures aimed at reducing the risks of introduction and exposure to AMR.
