# Supplementary material for: A qualitative approach for a situation analysis of AMR risks in the food animal production sector
Source: Front Vet Sci. 2023 Feb 16;10:1045276. doi: 10.3389/fvets.2023.1045276 (PMC9978409; doi:10.3389/fvets.2023.1045276)
Supplement: Supplementary file 1 [file Data_Sheet_1.PDF]

Supplementary Material

1 Annex 1. Instructions for the application of the survey for data collection for a situation analysis of AMR risks

The FAO situation analysis tool contains a document with instructions for the application of the survey, including sections on Introduction, Objectives, Instructions, Recommendations and Annexes with a glossary, the structure of the survey and the corresponding references.

Due to its extension, only the section on Recommendations is presented.

Recommendations

For the collection of information required by the Survey, it is essential to establish a Country Team for the Evaluation and Management of AMR Risks in animal production, with members who are able to contribute knowledge and experiences, from their respective disciplines, to complete the Survey. This group must be interdisciplinary, inter-institutional, inter-programmatic and include representatives of both the public and private sectors, as well as academia. Table 1 suggests key participants to address each component of the Survey. The participants should form a group of experts for each of the production systems and technical areas involved. Each group can manage the collection of information for Components 1, 2 and 3 independently. However, Component 4, which addresses cross-cutting elements that contribute to the risk of AMR, must be completed jointly by representatives of these different groups, incorporating the areas of expertise evaluated.

The process of filling out the Survey should include representatives of the National Intersectoral Committee on AMR, or at least the results of this initiative, led by the Agricultural Health and Food Safety Service, should be disseminated among the members of this committee.

Table 1. Suggested participants for each section of the Survey

Component	Suggested technical areas
1. Terrestrial animal production	National Coordinator<br>Representatives of:<br>Animal health (terrestrial)<br>Veterinary products and animal feed<br>Experts in terrestrial animal production (technology companies)<br>Industry organizations<br>Pharmaceutical industry<br>Food safety<br>Public health<br>Laboratories<br>Environment
2. Aquatic animal production	National Coordinator<br>Representatives of:<br>Animal health (aquatic)<br>Veterinary products and animal feed<br>Experts in aquatic animal production<br>Industry organizations<br>Pharmaceutical industry<br>Food safety<br>Public health<br>Laboratories

3. Family farming (terrestrial production and aquaculture)		National Coordinator<br>Representatives of:<br>Animal health (terrestrial and aquatic)<br>Veterinary products and animal feed<br>Public and private entities linked to agricultural development<br>Experts in family farming and food security<br>Food safety<br>Public health<br>Environment
4. Sustainability of the system	AMU and AMR surveillance	National Coordinator<br>Representatives of:<br>Animal health (terrestrial)<br>Animal health (aquatic)<br>Veterinary products and animal feed<br>Diagnostic laboratories (terrestrial and aquatic, official and private)<br>Food safety<br>Public health
	Institutional Governance of One Health and the Agrifood Sector	National Coordinator<br>Representatives of:<br>Public Health linked to the National Intersectoral Committee on AMR<br>Agrifood Sector linked to the National Intersectoral Committee on AMR<br>Authorities related to the environment<br>Legal Departments of Health and Agriculture<br>Animal health (terrestrial and aquatic)<br>Registration of veterinary products and animal feed<br>Academia
	Communication of risks (awareness, advocacy and training)	National coordinator<br>Representatives of:<br>Animal health (terrestrial and aquatic)<br>Food safety<br>Public health<br>Communication units of official health services<br>Experts in extension and health education<br>Experts in training and technical training<br>Industry organizations<br>Academia<br>Rural extension entities
	Research and innovation	National coordinator<br>Representatives of:<br>Animal health (terrestrial and aquatic)<br>Food safety<br>Public health<br>Academia (Universities and Research Centers)<br>Laboratories

As mentioned in the Instructions, it is very important to answer all the questions. Therefore, in the absence of exact information, the country is requested to provide estimates. There are several mechanisms or methods to obtain this type of information. Some examples are given below to facilitate these estimates in answering the corresponding questions in the Survey.

- Expert interviews: These consist of an information sharing activity that is guided in terms of the topics of interest. It is recommended to prepare a set of questions before the interview to obtain the required information. The interviews can be conducted in person, by phone or videoconference. It is recommended to obtain estimates from several experts (at least three) for each of the questions and provide an average to reduce uncertainty and possible biases in the answers.
- Focus group discussions: These consist of a group of several people interviewed at the same time for reasons of convenience (e.g. farmers from producer associations in a specific sector). This tool for

obtaining mainly qualitative information (although they can be used to give estimates of percentages for the group), depends both on the interaction or exchange of ideas among the participants, and on the answers to specific questions asked by the interviewer, or in this case the moderator. The moderator is responsible for guiding and facilitating the process. It is recommended to use this methodology with the support of experienced moderators to generate data and interpretations that would be less accessible without the group interaction (e.g. when biases related to social desirability are expected, especially in stakeholders that may have conflicts of interest).

- Surveys: These consist of the development of a set of questions related to a particular subject. These can be asked in person, by telephone or online. Regarding the latter, today there are free servers such as SurveyMonkey® or Google Forms, which can help to reach a large number of respondents quickly. The average estimates provided can be used to answer questions on the FAO Survey, when the sample size and characteristics of the respondents allow it, while controlling for biases and ensuring statistical significance.
- Expert elicitation process: This method involves the participation of several experts (at least three recommended) to obtain knowledge in order to resolve a problem, make a decision or achieve greater understanding of a particular problem. This tool can be applied remotely (questionnaire, telephone, other) or in person and a third party should supervise the process. The two main methods for categorizing the answers are: behavioural and mathematical. The first involves a group interaction, where experts share their assessments and knowledge to reach a consensus (e.g. Delphi method). The second may be an average of the estimates or involve a greater degree of difficulty including axiomatic and Bayesian techniques.

It is important to identify moderators with experience in the application of these tools, together with key respondents and experts in the areas related to the data required.

2 Annex 2. List of questions by survey component

Component 1: Terrestrial animals

- **Risk factor: Characterization of the production system**

1. By animal production system, indicate the animal population and the number of animal production farms categorize in large or medium scale (excluding Family Farming).
2. By animal production system, indicate the number of slaughterhouses, by type (municipal/public, commercial/ private).
3. By animal production system, indicate the percentage of animals slaughtered in each of the types of slaughterhouses.
4. Indicate the number of industrial producers of fresh and mature/aged cheese.
5. Indicate the volumes of national production, imports and exports of products of animal origin for human consumption (products: broiler meat, turkey meat, fresh eggs, pork, beef, sheep and goat meat, processed meat products (cured meat, sausages, others), pasteurized milk, fresh cheese, mature/aged cheese, fresh eggs).

- **Risk factor: Sanitary conditions in animal production**

6. By animal production system, indicate the three most prevalent diseases or endemic bacterial agents (parasites, if applicable) during a production cycle that are treated or prevented with antibiotics. For each of the agents listed indicate: if any is a notifiable disease to the competent authority in your country, the frequency of occurrence of the disease/condition, and the antibiotics used for the treatment/control/prevention of the disease/agent and its administration route.

- **Risk factor: Farming practices in animal production**

7. By animal production system, indicate the percentage of animal production farms that keep records of the animal population, including the entry and exit of animals.

Component 1: Terrestrial animals

8. By animal production system, indicate the percentage of animal production farms that apply a system for individual or batch/flock identification of the animals. If yes, indicate if the registration system is official or private.
9. By animal production system, indicate the percentage of animal production farms that apply an animal identification system that allow traceability of animals or batches/flocks throughout the entire production process within the same farm.
10. By animal production system, indicate the percentage of animal production farms that use records to register diseases and infections detected during the productive cycle.
11. By animal production system, indicate the percentage of animal production farms that use morbidity records.
12. By animal production system, indicate the percentage of animal production farms that use mortality records.
13. By animal production system, indicate the percentage of animal production farms that keep records of animal treatments and procedures (castration, vaccinations, treatment application, among others).
14. By animal production system, indicate the percentage of animal production farms that have veterinary assistance or another professional or technician recognized by the competent authority for the care of production animals.
15. By animal production system, indicate the type of professional (s) and/or agricultural technician (s) with legal attributions to perform an animal clinical diagnosis.
16. On the animal production farm, indicate what percentage of the clinical diagnoses of diseases/illnesses are made by the following actors linked to the production process: veterinarian, veterinary/agricultural technician, supervisor, animal caretaker, other (indicate) (in aquaculture: veterinarian, aquaculture engineer or marine biologist, veterinary/aquaculture technician, supervisor, animal caretaker, other (indicate).
17. By animal production system, indicate the percentage of the level of application of internal biosecurity measures to prevent the spread of infectious agents within the animal production farm.
18. By animal production system, indicate the percentage of application of external biosecurity measures to prevent the entry and dissemination of infectious agents to and from animal production farms.
19. By animal production system, indicate the percentage of the level of application of measures to ensure animal welfare on the farm.

- **Risk factor: Practices of antibiotics use in food producing animals**

20. By animal production system, indicate the professional (s) or agricultural technician (s) with legal attributions to make the decision to use antibiotics and prescribe them. In case it is not established, please indicate.
21. Indicate the percentage of the following suppliers of veterinary antibiotics used by animal production farms: establishment regulated and authorized by the competent authority, establishment NOT regulated or authorized by the competent authority.
22. By animal production system, indicate the percentage of use of the following antibiotic administration routes. For this question, consider all applications made (independent of their purpose).
23. By animal production system, indicate what percentage of the production system depends on the use of antibiotics. For this answer, do not consider the use of antibiotics as therapeutic treatment.

(Questions 24-37 are answered excluding the application of antibiotics through feed).

24. By animal production system, indicate the percentage of animal production farms where the decision to use antibiotics is made by the following actors linked to the production process: veterinarian, veterinary/agricultural technician, supervisor, animal caretaker, other (in aquaculture: veterinarian, aquaculture engineer/marine biologist, veterinary/aquaculture technician, supervisor, other).
25. Of the factors listed below, by animal production system, indicate the five most used to decide the application of antibiotics. Order your answer from highest to lowest. Factors: (a) Clinical diagnosis, (b) Laboratory diagnosis to determine the infectious agent, (c) Experience, (d) History of diseases and

pathogens in the animal production farm, (e) Levels of morbidity, (f) Levels of mortality, (g) Cost of application, (h) Availability on the animal production farm, (i) Other (indicate).

26. Of the factors listed below, by animal production system, indicate the five most used to decide which antibiotics to apply. Order your answer from highest to lowest. Factors: (a) Clinical diagnosis, (b) Laboratory diagnosis to determine the infectious agent, (c) Experience, (d)History of diseases and pathogens in the animal production farm, (e) Levels of morbidity, (f) Levels of mortality, (g) Cost of the product to be used, (h) Causative agent sensitivity tests, (i) Pharmacokinetic and pharmacodynamic characteristics of the product to be used, (j) Effectiveness, (k) Withdrawal period, (l) Seller recommendations, (m) Market trends, (n) Availability in the market, (o) Custom, (p) List of critically important antibiotics from WHO and OIE, (q) Impact on public health, (r) Recommendations of family or friends, (s) Other (indicate).
27. By animal production system, indicate the percentage of animal production farms that apply antibiotics with a prescription from the legally responsible professional (indicated in question 20). If there is no legal responsible designated, indicate the percentage of application with the support of a veterinary prescription.
28. By animal production system, indicate the percentage of those who prescribe antibiotics do so leaving instructions for their application (dose, application interval and duration of treatment), route of administration and withdrawal period (if applicable).
29. By animal production system, indicate the percentage of animal production farms that follows the manufacturer's recommendations for the application of antibiotics (indicated on the product labelling/competent authority registration records).
30. By animal production system, indicate the percentage of animal production farms that use antibiotics as therapeutic treatment, preventive treatment (prophylactic and/or metaphylactic) and growth promoter. For those species where antibiotics are not used for one or more of the stated purposes, answer does not apply (N/A).
31. By animal production system, indicate the main person responsible for carrying out the application of antibiotics in animals (terrestrial animals: veterinarian, veterinary/agricultural technician, supervisor, animal caretaker, or other; aquaculture: veterinarian, aquaculture engineer, marine biologist, veterinary / aquaculture technician, supervisor, animal caretaker, or other)
32. For the person responsible indicated in the previous question, indicate the percentage that person is qualified for the application of antibiotics.
33. By animal production system, indicate the percentage of animal production farms that use antibiotics authorized by the competent authority.
34. By animal production system, indicate the percentage of animal production farms that maintain records of the application of antibiotics that includes, at least: animal or lot to which it has been applied, antibiotic used, quantity applied, dose applied, duration of application, date of application, days of withdrawal period if applicable.
35. By animal production system, indicate the percentage of animal production farms that comply the withdrawal period following the application of antibiotics (declared by the manufacturer/competent authority).
36. By animal production system, indicate the percentage of animal production farms whose antibiotics are stored following the manufacturer's recommendations to avoid deterioration, such as: temperature, direct light and humidity.
37. By animal production system, indicate the percentage of animal production farms whose antibiotics are used respecting the expiration date indicated in the product labelling.

- **Risk factor: Feed practices**

38. Indicate the number of importers, producers and sellers of medicated and non-medicated concentrate feeds. If the same establishment fulfils more than one function, indicate in the observations section.
39. By animal production system, indicate the quantity of concentrate feeds for animal use produced, imported and sold in the country. For each category, indicate the amount or percentage of feeds that meet the characteristic of being medicated with antibiotics.
40. Indicate the percentage of medicated concentrate feeds with erroneous or misleading labelling regarding their antibiotic content that prevents producers from knowing if they are administering antibiotics in feed.

Component 1: Terrestrial animals

41. By animal production system, indicate the percentage of concentrate feeds producers that follow procedures to minimize physical and microbiological contamination.
42. By animal production system, indicate the percentage of producers of medicated and non-medicated concentrate feeds in independent production chains.
43. By animal production system, indicate the percentage of concentrate feeds that is transported and distributed following procedures to minimize physical and microbiological contamination.
44. By animal production system, indicate the percentage of concentrate feeds producers that have traceability processes.
45. By animal production system, indicate the percentage of animal production farms that have management processes to prevent physical and microbiological contamination in concentrate feeds handling and storage.
46. By animal production system, indicate the percentage of animal production farms that maintain an auditable record of concentrate feeds used, which includes background information such as: processing date, expiration date, target animals, among others. In the case of feed mixed on the farm, if a record of its ingredients and mixtures, dates of consumption, expiration date, animals of destination, among others, is maintained.
47. By animal production system, indicate the percentage of drinking water (high-quality water that can be consumed or used without risk) that is used for animals.
48. By animal production system, indicate the origin of the medicated concentrate feeds (as a percentage): self-producer, commercial producer (national, imported).
49. By animal production system, indicate the percentage of animal production farms where the decision to use antibiotics through concentrate feeds is made by the following actors linked to the production process: veterinarian, veterinary/agricultural technician, supervisor, animal caretaker, other (indicate) (in aquaculture: Veterinarian, aquaculture engineer or marine biologist, veterinary/aquaculture technician, supervisor, caretaker, other).
50. Of the factors listed below, by animal production system indicate the five most commonly used to determine the use of antibiotics in concentrate feeds. Order your answers from highest (most important) to lowest (least important). Factors: (a) Clinical diagnosis, (b) Laboratory diagnosis to determine the infectious agent, (c) Experience, (d) History of diseases and pathogens in the animal production farm, (e) Levels of morbidity, (f) Levels of mortality, (g) Cost of application, (h) Availability on the animal production farm, (i) Other (indicate).
51. Of the factors listed below, by animal production system indicate the five most commonly used to determine the use of antibiotics in concentrate feeds. Order your answers from highest (most important) to lowest (least important). Factors: (a) Clinical diagnosis, (b) Laboratory diagnosis to determine the infectious agent, (c) Experience, (d)History of diseases and pathogens in the animal production farm, (e) Levels of morbidity, (f) Levels of mortality, (g) Cost of the product to be used, (h) Causative agent sensitivity tests, (i) Pharmacokinetic and pharmacodynamic characteristics of the product to be used, (j) Effectiveness, (k) Withdrawal period, (l) Seller recommendations, (m) Market trends, (n) Availability in the market, (o) Custom, (p) List of critically important antibiotics from WHO and OIE, (q) Impact on public health, (r) Recommendations of family or friends, (s) Other (indicate).
52. By animal production system, indicate the percentage of establishments where the preparation of medicated concentrate feeds is carried out with a prescription from the legally responsible professional (indicated in question 20). If there is no legal designation, indicate the percentage of feed produced with a veterinary medical prescription.
53. By animal production system, indicate what percentage of professionals responsible for prescribing medicated concentrate feeds provide instructions for its application (dose, application interval and duration of treatment), route of administration and withdrawal period (if applicable).
54. By animal production system, indicate the percentage of animal production farms that follow the manufacturer's recommendations (indicated in the product labelling or authorization document or registration of the competent authority) for the application of antibiotics by means of medicated concentrate feeds.
55. By animal production system, indicate the percentage of animal production farms that use medicated concentrate feeds as therapeutic treatment, preventive treatment (prophylactic and/or metaphylactic) and

as growth promoter. For those species where antibiotics are not used for one or more of the stated purposes, answer does not apply (N/A).

56. By animal production system, indicate the main person responsible for carrying out the application of medicated concentrate feeds (veterinarian, veterinary/agricultural technician, supervisor, animal caretaker or other; in aquaculture: veterinarian, aquaculture engineer, marine biologist, veterinary/aquaculture technician, supervisor, animal caretaker or other).
57. For the person responsible in the previous question, indicate (as a percentage) the level of their technical capabilities and competencies to carry out the application of medicated concentrate feeds.
58. By animal production system, indicate the percentage of use of antibiotics authorized and registered by the relevant authority in the preparation of medicated concentrate feeds.
59. By animal production system, indicate the percentage of animal production farms that maintain a record of the application of medicated concentrate feeds that includes: animals or batch of animals, antibiotics used, amount, dosage, duration, date of application, and withdrawal period if applicable.
60. By animal production system, indicate the percentage of animal production farms that comply with the withdrawal period after the application of medicated concentrate feeds (stated by the manufacturer).
61. By animal production system, indicate the number of producers of medicated concentrate feeds that have a traceability mechanism from production to their use on the animal production farm.
62. By animal production system, indicate the percentage of medicated concentrate feeds that are kept in conditions that do not affect their stability and prevent contamination during transport.
63. By animal production system, indicate the percentage of medicated concentrate feeds that are stored under conditions that do not affect their stability and prevent contamination.

- **Risk factor: Environmental management practices**

64. By animal production system, indicate the percentage of animal production farms that use the following guano disposal methods: fertilizer for agricultural production with previous treatment, fertilizer for agricultural production without previous treatment, bodies of water with previous treatment, bodies of water without previous treatment, compost, burial, energy generation (biomass), other (indicate).
65. By animal production system, indicate the percentage of animal production farms that use the following slurry disposal methods: fertilizer for agricultural production with previous treatment, fertilizer for agricultural production without previous treatment, bodies of water with previous treatment, bodies of water without previous treatment, compost, burial, energy generation (biomass), other (indicate).
66. By animal production system, indicate the percentage of animal production farms that use the following methods for the disposal of dead animals: rendering, burial, sealed pit, domestic waste, bodies of water, incineration, other (indicate) (in aquaculture: rendering, burial, sealed pit, silo, domestic waste, bodies of water, incineration, other (indicate).
67. By animal production system, indicate the percentage of animal production farms that use the following methods for the disposal of medicated concentrate feeds: feed, compost, domestic waste, bodies of water, other (indicate), not eliminated.
68. By animal production system, indicate the percentage of animal production farms that use the following methods for the disposal of antibiotics: domestic waste, collected by a specialized company, bodies of water, burial, other (indicate), not eliminated.
69. By animal production system, indicate the percentage of animal production farms that use the following methods for the disposal of antibiotics containers: domestic waste, collected by a specialized company, bodies of water, burial, other (indicate).
70. By animal production system, indicate the percentage of feed concentrate producers that use the following methods for the disposal of medicated feed: feed, compost, domestic waste, sold to third parties (commercial), other (indicate), not eliminated.
71. By animal production system, indicate the percentage of concentrate feeds producers that use the following methods for the disposal of antibiotics: domestic waste, collected by a specialized company, bodies of water, burial, other (indicate), not eliminated.
72. By animal production system, indicate the percentage of concentrate feeds producers that use the following methods for the disposal of antibiotics containers: domestic waste, disposal by a specialized company, bodies of water, burial, other (indicate).

73. By animal production system, indicate the percentage of slaughterhouses that use the following methods for the disposal of dead animals: domestic waste, rendering, bodies of water, burial, sold to third parties, other (indicate).
74. By animal production system, indicate the percentage of slaughterhouses that use the following methods for disposal of waste and remains: domestic waste, rendering, bodies of water, burial, sold to third parties, other (indicate).

- **Risk factor: Direct contact with food-producing animals and animal products**

75. By animal production system, indicate the number of people nationwide who works on animal production farms. Consider only those people who are in direct contact with the animals (excluding family farming).
76. By animal production system, indicate the number of people working in slaughterhouses and processing plants that process food of animal origin for human consumption. Consider only those people who are in direct contact with products of animal origin.

- **Risk factor: Consumption of food of animal origin**

77. By animal production system, indicate the domestic consumption per capita (kg/person/year) according to the type of product of animal origin (products: broiler meat, turkey, fresh eggs, pork, beef, sheep-goat meat, processed meat products -cured meat, sausages, others-, pasteurized milk, fresh cheese, mature/aged cheese).
78. Indicate the percentage of people who eat outside home at least once a week.
79. By type of product, indicate the trend of consumption of products of animal origin inside and outside the home (food product: broiler meat, turkey, pork, beef).
80. In terms of food consumption inside and outside the home, indicate the percentage of food that is consumed raw or cooked (food product: broiler meat, turkey, pork, beef).

Mitigation measures associated with the production of terrestrial animals

Each legislation related question must be answered including the following parameters:

- *Existence: indicate the existence of legislation on the subject.*
- *Quality of legislation: answer yes if the legislation is well designed to meet its objective, and no if the objective is not met.*
- *Coverage: indicate whether the legislation applies at the national level, or only to a specific group.*
- *Quality of application: indicate if the legislation is applied correctly. Submit your answer in Percentage.*
- *Difficulties of application: indicate whether the application of the legislation is compromised by deficiencies in economic resources and/or human capital.*

- **Risk factor: Characterization of the production system**

81. Legislation related questions:

Legislation exists in animal production, which includes:

- Requirements for the identification and registration/authorization of animal production farms and their animals.
- Measures that ensure animal traceability from the animal production farm to the slaughterhouse.

- **Risk factor: Sanitary conditions in animal production, farming practices in animal production**

82. By animal production system, indicate the percentage of application of public and/or private programs for the implementation of good animal production practices.
83. Regarding the above, does the competent authority conduct regular verification processes?
84. Regarding the infectious agents mentioned in question 6, by animal production system indicate the existence and percentage of vaccine application among the animal production farms.

Component 1: Terrestrial animals

85. Legislation related questions:

Legislation exists for professional(s) and/or technician(s), which:

- Defines the responsibilities and attributions for the diagnosis of animal diseases.

Legislation exists for animal production, which establishes:

- Requirements for the identification and registration/authorization of animal production farms and their animals.
- A surveillance system (collection, transmission and use of epidemiological data) of endemic bacterial agents of importance to the country.
- Sanitary measures for the prevention of diseases in production animals such as: good production practices and biosecurity.
- Sanitary measures for the control of diseases in production animals such as: good production practices and biosecurity.
- The minimum measures to ensure animal welfare on animal production farms.
- Measures that ensure animal traceability from animal production farms to the slaughterhouse.

- **Risk factor: Practices of antibiotics use in food producing animals**

86. In animal production, indicate the level of existing knowledge regarding the use of antibiotic alternatives: veterinarian, veterinary/agricultural technician, supervisor, animal caretaker, other (indicate) (aquaculture: veterinarian, aquaculture engineer or marine biologist, veterinary/aquaculture technician, supervisor, caretaker, other).
87. In animal production, what is the level of application of the following products as alternatives to the use of antibiotics? (Alternatives: prebiotics, probiotics, organic acids, bacteriophages, vitamin supplements, natural extracts, other).
88. Legislation related questions:

Legislation exists on veterinary antibiotics, which establishes:

- The professional(s)/technician(s) with legal attributions to issue prescriptions for veterinary antibiotics.
- The definition of veterinary antibiotics.
- The requirements for the import, production, commercialization and distribution of antibiotics for use in production animals.
- The requirements for the use of antibiotics in production animals.
- Registration or authorization of businesses involved in the production, importation and sale of veterinary antibiotics.
- The quality requirements for the raw material used in the manufacture or production of veterinary antibiotics.
- The conditions for the approval, registration, renewal, rejection and cancellation of authorizations for veterinary antibiotics.
- The exclusive sale of veterinary antibiotics by establishments authorized by the competent authority.
- Conditions to ensure the effectiveness of veterinary antibiotics, such as: laboratory studies, clinical trials, others.
- The conditions to ensure the safety of veterinary antibiotics, such as: toxicological studies, environmental assessment, waste management, others.
- The use of good practices by establishments involved in the production, storage or sale of veterinary antibiotics.
- The presence of a veterinarian or other qualified professional with technical responsibility in establishments involved in the production and sale of veterinary antibiotics.
- Measures for traceability and the withdrawal of veterinary antibiotics from market.
- The requirements for the prescription and supply of veterinary antibiotics to end users.

- The requirements for the sale of antibiotics only with a prescription from a veterinarian or other professional or technician with legal attributions.
- The requirements to respect the withdrawal period of veterinary antibiotics in production animals.
- The maximum residual limits of veterinary antibiotics in foods of animal origin for human consumption.
- The prohibition of the use of antibiotics as growth promoters.
- The prohibition of the use of antibiotics as prophylactic or preventive treatments.
- The control of advertising and other promotional and marketing activities.
- The requirement for a pharmacovigilance system in the country.

- **Risk factor: Feed practices**

89. By animal production system, are there public or private programs for the application of good practices in the production of concentrate feeds? Give you answer as a percentage corresponding to the level of implementation.
90. Regarding the above, are there regular verification processes by the competent authority?
91. By animal production system, are there public or private programs for the application of HACCP in the production of concentrate feeds? Give you answer as a percentage corresponding to the level of implementation.
92. Regarding the above, are there regular verification processes by the competent authority?
93. For each animal production system, are there public or private programs for the detection of antibiotic residues in concentrate feeds?
94. Regarding the above, are there regular verification processes by the competent authority?
95. For each animal production system, are there public or private programs for the detection of bacterial contaminants in concentrate feeds?
96. Regarding the above, are there regular verification processes by the competent authority?
97. Legislation related questions:

Legislation exists on concentrate feeds, which establishes:

- Registration of establishments authorized by the relevant authority for the production of concentrate feeds, whether these are self-processors or commercial processors.
- Registration of establishments involved in the import of concentrate feeds that are authorized/approved by the relevant authority.
- Regulations for the production, composition and quality control of concentrate feeds (including good production practices).
- Regulations that require producers to use good practices to prevent cross contamination of concentrate feeds.

Legislation exists on medicated concentrate feeds, which establishes:

- The production of medicated concentrate feeds only with a prescription issued by a professional with legal attributions, or by a veterinarian.
- The standards of production, composition and quality control of medicated concentrate feeds (including good production practices).
- A prohibition on the addition of antibiotics to concentrate feeds for the purpose of growth promotion.
- A prohibition on the addition of antibiotics to concentrate feeds for preventive purposes.
- The exclusive use of antibiotics authorized and registered by the relevant authority for the production of medicated concentrate feeds.

- **Risk factor: Environmental management practices**

98. By animal production system, are there public or private programs for the application of good environmental management practices in animal production? Give you answer as a percentage corresponding to the level of implementation.
99. Regarding the above, are there regular verification processes by the competent authority?
100. Legislation related questions:

Component 1: Terrestrial animals

Legislation exists for the final disposal of waste during the animal production chain, which establishes:

- Measures for the disposal of organic (including slurry), chemical and physical waste in animal production farms.
- Measures for the elimination of organic, chemical and physical waste by feed producers.
- Measures for the disposal of organic (including slurry), chemical and physical waste in slaughterhouses and processing plants that process food of animal origin for human consumption.

- **Risk factors: Consumption of food of animal origin, Consumption of food of animal origin contaminated with antimicrobial residues, Consumption of food of animal origin contaminated with bacteria, Direct contact with food-producing animals and animal products** (the information obtained from this questions allows to evaluate risks and mitigation measures)

101. For each product of animal origin for human consumption (chicken, turkey, pork, beef, sheep-goat meat, processed meat products -cured meat, sausages, others-, fresh eggs, milk, fresh cheese, mature/aged cheese, other dairy products -milk powder, yoghurt, others-, honey), indicate whether your country has programs for the detection and control of antibiotic residues in foods of animal origin for human consumption. If such a program exists, indicate: If it is managed by the public health authority (Agriculture or Public Health), by the private sector or both; the product category in which the analysis is carried out (national production (national consumption), imported, for export); location where the program is implemented (animal production farm, slaughterhouse, processing plant).
102. Regarding the analyses performed for the detection of antibiotic residues in products of animal origin, indicate the total number of samples taken and the number of results obtained above the maximum residual limits (MRLs) allowed in your country.
103. Regarding the results obtained, indicate whether these are shared publicly. If yes, indicate if these are communicated to a National AMR Program.
104. For each product of animal origin for human consumption (chicken, turkey, pork, beef, sheep-goat meat, processed meat products -cured meat, sausages, others-, fresh eggs, milk, fresh cheese, mature/aged cheese, other dairy products -milk powder, yoghurt, others-, honey), indicate whether your country has microbiological control programs for foods of animal origin for human consumption. If such a program exists, indicate: If it is managed by the public health authority (Agriculture or Public Health), or by the private sector; Product category to which analysis is carried out (national production, imported, for export); location where the program is implemented (animal production farm, slaughterhouse, processing plant, other).
105. Regarding the analyses performed for microbiological detection in products of animal origin for human consumption, indicate: total number of samples analysed; number of positive samples according to the type of bacteria. A positive sample is considered to be one whose bacterial load exceeds that established by the competent authority.
106. Indicate if your country has training programs in good practices to prevent microbiological contamination in establishments where food for human consumption is prepared, for example: restaurants, cafes, others.
107. Indicate if your country has awareness raising activities on safe food handling practices at home.
108. Legislation related questions:

Legislation exists related to laboratories, which establishes:

- The responsibilities, obligations, quality levels and control of reference laboratories.
- The responsibilities, obligations, quality levels and control of non-reference laboratories.

Legislation exists regarding the production of food for human consumption, which establishes:

- Requirements to ensure the health of workers in the livestock area.
- The registration of slaughterhouses.
- The registration of processing plants that process food of animal origin for human consumption.
- Sanitary norms for the infrastructure, equipment and operation of slaughterhouses.
- Sanitary norms for infrastructure, equipment and operation of processing plants that process food of animal origin for human consumption.
- Health inspection standards for slaughterhouses.

Component 1: Terrestrial animals

- Health inspection standards for processing plants that process food of animal origin for human consumption.
- Standards for the audit of slaughterhouses.
- Standards for the audit of processing plants that process food of animal origin for human consumption.
- The promotion and/or application of safety assurance systems (such as HACCP) in slaughterhouses.
- The promotion and/or application of safety assurance systems (such as HACCP) in processing plants that process food of animal origin for human consumption.
- Measures for monitoring and control (official or private) of foodborne diseases and/or bacterial agents in slaughterhouses.
- Measures for monitoring and control (official or private) of foodborne diseases and/or bacterial agents in processing plants that process food of animal origin.
- Measures for monitoring and control (official or private) of antibiotic residues in slaughterhouses.
- Measures for the monitoring and control (official or private) of antibiotic residues in processing plants that process food of animal origin for human consumption.
- Requirements for antibiotic residues in imported foods of animal origin for human consumption.
- Requirements for bacterial contaminants in imported foods of animal origin for human consumption.
- Requirements for traceability in slaughterhouses.
- Requirements for traceability in processing plants that process food of animal origin for human consumption.
- Food safety requirements (for products of national origin) for products of animal origin destined for human consumption during the commercialization process.
- Food safety requirements (for imported products) for products of animal origin destined for human consumption during the commercialization process.

Component 2: Aquatic animals

Excluding the questions 4, 47, 64 and 78, the questions in the Aquatic Animals component are the same as in component 1.

Component 2 includes questions 109 to 212.

Component 3: Family farming

Questions referring to family farming groups animal production systems: terrestrial and aquatic.

Risk factors associated with the production of animals

- **Risk factor: Characterization of the production system**

Family farming is defined as: Animal production (terrestrial and aquaculture), managed and run by a family and which mostly depends on family labour (women, children and men) (Definition adapted from: http://www.fao.org/family-farming-decade/about/en).

213. Considering that the definition of family farming varies geographically and culturally, adapt the given definition to such a way that it is the most representative of the sector in your country.

214. Based on what is understood by the concept of family farming, indicate the number of farms for the production of terrestrial and aquatic species that are present in your country.

215. By animal production system, indicate the total number of animals produced in family farming. If you do not know the number, indicate the percentage of the total animal population of the country to which it corresponds.

216. Describe the structure of the most common types of family farms in your country (animal species, size, geographic areas, product distribution chains and participation of the local community, etc.). In addition to this, indicate the current and projected trends in the growth of family farming in your country in recent years.

Given that Family farming is generally dedicated to the breeding of multiple animal species, if necessary, the questions below can be answered considering the predominant species for this model of productive system in your country.

217. For terrestrial and aquatic animals, indicate the facilities where these animals are processed or slaughtered (family farm, private, municipal/public, sold to third parties).

218. Indicate the number of fresh and mature artisanal cheese producers.

- **Risk factor: Sanitary conditions in animal production**

219. Indicate, by group of animal production systems, the three most prevalent diseases or endemic bacterial agents (parasites, if applicable) during a production cycle that are treated or prevented with antibiotics. For each of the agents listed indicate: if any is a notifiable disease to the competent authority in your country, the frequency of occurrence of the disease/condition, and the antibiotics used for the treatment/control/prevention and its administration route.

- **Risk factor: Farming practices in animal production**

220. Indicate the percentage of family farming farms that carry out animal identification (individual/ lot/flock).

221. Indicate the percentage of family farming farms that record the animal population.

222. Indicate the percentage of family farming farms that register detected diseases.

223. Indicate the percentage of family farming farms that record animal mortalities.

224. Indicate the percentage of family farming farms that record animal treatments and procedures (castration, vaccinations, product application, others).

225. Indicate the percentage of family farming farms that use the following biosecurity measures, focused on preventing the entry and dissemination (inside and outside the farm) of infectious agents:
    - periodic cleaning of facilities where animals are kept;
    - application of products for disinfection of facilities post-cleaning;
    - periodic collection of guano;
    - cleaning stations for disinfecting hands and shoes;
    - removal of dead animals in the shortest possible time;
    - control of pests, such as rodents and insects;
    - separation of healthy and sick animals;
    - use of special clothing and/or footwear exclusively for productive activities;
    - food handling in conditions that prevent contamination;
    - separation of animal production from agricultural activities.

226. Indicate the percentage of family farming farms that have veterinary assistance, or services of other professionals or technicians recognized by the competent authority for the care of farm animals.

227. In a family farming farms, indicate what percentage of the clinical diagnoses of disease/illnesses are made by the following actors linked to the productive process: professional with legal attributions, veterinarian, veterinary/agricultural technician, supervisor, animal caretaker, other (indicate). If there is no professional with legal attributions, indicate what percentage of the clinical diagnoses of disease/illnesses are made by a veterinarian, veterinary/agricultural technician, supervisor, animal caretaker or other (indicate).

- **Risk factor: Practices of antibiotics use in food producing animals**

228. Indicate, as a percentage, the percentage of the following suppliers of veterinary antibiotics used by family farming: establishment regulated and authorized by the competent authority, establishment not regulated or authorized by the competent authority.

229. In what percentage the decision of applying antibiotics is made by a veterinarian, veterinary/agricultural technician, owner/relative, and animal caretaker.
230. In what percentage the antibiotic prescription are made by a veterinarian, veterinary/agricultural technician, owner/relative, animal caretaker, other (indicate).
231. Indicate the percentage of the prescriptions that are accompanied by indications for the antibiotics application (dosage, application interval and duration of treatment), route of administration and withdrawal period (if applicable).
232. In the family farming, indicate what percentage of antibiotic applications are carried out with a prescription given by a professional with legal attributions or a veterinarian.
233. In case of using a prescription for the application of antibiotics in family farming, indicate the percentage with which the indications are applied.
234. Indicate the percentage of family farms that use damaged, expired or contaminated antibiotics.
235. Indicate the percentage of family farms that follow the manufacturer's recommendations for the application of antibiotics ((indicated on the product labelling/competent authority registration records).
236. Indicate the percentage of family farms that use antibiotics as therapeutic treatment, preventive treatment (prophylactic and/or metaphylactic) and as a growth promoter. For those animal production systems where antibiotics are not used in one or more of the stated purposes, please answer not applicable (N/A).
237. Indicate the percentage of family farms that use antibiotics authorized by the competent authority.
238. Indicate the percentage of family farms that comply with the withdrawal periods after the application of antibiotics.

- **Risk factor: Feed practices**

239. Indicate the percentage of family farms that use concentrate feeds.
240. If used, indicate the origin of the concentrate feeds.
241. Of the family farms that use concentrate feeds, what percentage of them use medicated feed with antibiotics?
242. Indicate the percentage of medicated concentrated feeds with erroneous or misleading labelling regarding their antibiotic content that prevents family farmers from knowing if they are administering antibiotics in feed.

- **Risk factor: Environmental management practices**

243. Indicate the percentage of family farms that use the following guano disposal methods: fertilizer for agricultural production, grassland fertilizer, compost, bodies of water, burial, animal feed, other (indicate).
244. Indicate the percentage of family farms that use the following methods for the disposal of dead animals: domestic waste, bodies of water, burial, consumption, consumption by other animals, incineration, not eliminated, other (indicate).
245. Indicate the percentage of family farms that use the following methods for the disposal of antibiotics: domestic waste, bodies of water, burial, incineration, used despite being expired, not eliminated.
246. Indicate the percentage of family farms that use the following methods for the disposal of antibiotic containers: domestic waste, bodies of water, burial, incineration, specialized disposal, other (indicate).

- **Risk factors: Direct contact with food-producing animals and animal products, Consumption of food of animal origin**

247. Indicate the total number of people, at the national level, who work on family farms (terrestrial and aquatic). If you do not know the exact number, indicate the average number of people, whether women, men or children, who work on family farms.
248. Of the products listed below (poultry, pork, beef, fish, crustaceans, meat of other species, processed meat products, fresh eggs, milk, dairy products, honey, seafood, other), what percentage comes from family farming?
249. Of the products produced by family farming (poultry, pork, beef, fish, crustaceans, meat of other species, processed meat products, fresh eggs, milk, dairy products, honey, seafood, other), indicate the percentage destined for consumption within the family nucleus.
250. By type of product (terrestrial species, aquatic species), indicate the trend of consumption of products of animal origin inside and outside the home.
251. For the food consumed inside and outside the home, indicate the percentage that is consumed raw or cooked.

Mitigation measures associated with family farming

Part of the information gathered in components 1 and 2 is also used to evaluate the mitigation measures in family faming.

- **Risk factors: Characterization of production system, Farming practices in animal production**

252. Regarding the endemic infectious agents mentioned in question 219, by group of animal production systems, indicate the existence and percentage of vaccination applied in family farming.
253. The country has programs for the promotion of family farming (public, private, public/private).
254. The country has programs for the application of biosecurity and sanitary management measures for family farming (whether these are specific to biosecurity/sanitary management or are incorporated into a Program: public, private, and public/private).

- **Risk factor: Practices of antibiotics use in food producing animals**

255. The country has family farming programs for the appropriate use of antibiotics (whether these are specific to the use of antibiotics or that incorporate it as part of the Program) (Public, private, public/private).
256. In family farming, what is the level of application of the following products as alternatives to the use of antibiotics? (Prebiotics, probiotics, organic acids, vitamin supplements , natural extracts, others).

Component 4: System sustainability factors

Each legislation related question must be answered including the following parameters:

- *Existence: indicate the existence of legislation on the subject.*
- *Quality of legislation: answer yes if the legislation is well designed to meet its objective, and no if the objective is not met.*
- *Coverage: indicate whether the legislation applies at the national level, or only to a specific group.*
- *Quality of application: indicate if the legislation is applied correctly. Submit your answer in Percentage.*
- *Difficulties of application: indicate whether the application of the legislation is compromised by deficiencies in economic resources and / or human capital.*

- **Factor: Antibiotic use surveillance**

257. Indicate the number of establishments for the national production, import and sale of antibiotics for veterinary use. If the same establishment fulfils more than one function, indicate in the observations section.
258. Using your national results from the WOAH questionnaire on the use of antimicrobial agents in animals, indicate, according to the type of terrestrial or aquatic animal production and total animal species, the national sales volume for each of the classes of antibiotics listed. Alternatively, you can attach the last completed WOAH questionnaire. If you do not have the WOAH questionnaire, answer the following question: by animal production system, indicate the 5 most used antibiotics. For each of them, estimate the annual amount used or the percentage it represents of the annual total used.
259. By animal production system, indicate if your country has a monitoring program (s) on the use of antibiotics in animal production. For each one, give a brief description (person responsible, objective, year launched, scope, other) and indicate if the information generated is shared with official institutions.
260. By animal production system, indicate if your country has a pharmacovigilance program (s) for the use of antibiotics in animal production. For each one, give a brief description (person responsible, objective, year of initiation, scope, other) and indicate if the information generated is shared with official Institutions.

261. In addition to the existence of programs, does the country have communication or contact mechanisms with the livestock/aquaculture industry to receive complaints or other situations related to pharmacovigilance? If yes, provide a brief description of the process.
262. By animal production system, indicate (in percentage) the frequency with which those responsible for animal farms (terrestrial/aquatic), if necessary, report failures in antibiotic treatments to the competent authority.
263. Legislation related questions:

Legislation exists that:

- Establishes a monitoring program on the use of antibiotics in animal production (terrestrial and aquatic).
- Establishes a pharmacovigilance program in animal production (terrestrial and aquatic).

- **Factor: AMR surveillance**

264. Does the country have laboratories that perform antibiotic sensitivity tests? By type of laboratory (public or private – academy/research), indicate the number of laboratories that perform antibiotic sensitivity tests in your country. For each one, indicate if the laboratory process samples of human origin, animal origin or non-specific.
265. Identify the number of reference laboratories for antibiotic sensitivity testing. For each one, indicate if the laboratory process samples of human origin, animal origin or non-specific.
266. Indicate if your country has an articulated network for the identification of AMR in public health and animal health. If yes, identify the participants.
267. Indicate if your country has applied the FAO ATLASS tool. If yes, indicate the current level of your country.
268. Indicate if your country has antibiotic resistance monitoring programs. In relation to the AMR monitoring program(s), indicate: responsible institution, type (public, private), year launched, origin of the analysed sample (food for animal use, animal intended for food, food of animal origin for human consumption), average number of samples analysed in one year, technique used for bacterial identification (for example, biochemical tests, automated method, miniaturized methods, MALDI-TOF, others), technique used for the diagnosis of AMR (disk diffusion, MIC, automated, gradient strip, other) and the application of proficiency tests.
269. In terms of the institutions indicated in the previous question, identify your contact and the associated laboratories (name and contact).
270. In relation to the AMR diagnostic techniques used in the monitoring program(s), indicate the international standard used for the performance and interpretation of sensitivity tests (for example: EUCAST, CLSI, other) and its year of publication. Use the institution responsible for program identification.
271. In relation to AMR monitoring programs, AMR diagnostic methods are evaluated using reference strains (ATCC).
272. In relation to AMR monitoring programs, the laboratory applies quality assessments.
273. In relation to AMR monitoring programs, the human capital responsible for the analysis and interpretation of results has been trained for this activity.
274. In relation to AMR monitoring programs, the analysed samples and their results are preserved and recorded.
275. In relation to AMR monitoring programs, do they have human capital and material resources for their proper functioning?
276. The results obtained from the AMR monitoring program(s) are delivered to the specialized unit designated by the country's competent authority. The results obtained from the AMR monitoring program(s) are analysed by the specialized unit.
277. In relation to the results obtained from the AMR monitoring program(s), these are analysed in an integrated manner (human health, veterinary health, environmental health).
278. The results obtained from the AMR monitoring program(s) are delivered in a periodic report. If yes, indicate whether these is public or aimed at specialized personnel.
279. Periodic reports are made under an integrated approach (human health, veterinary health, environmental health).
280. There is evidence of AMR in animal production, food of animal origin for human consumption or in human health related to animal production? If yes, indicate the person responsible for the results

(academia, private entity, public agency, other), origin of the positive sample (human, food of animal origin, animal production), resistant agent detected, date of information.

281. Legislation related questions:

Legislation exists that:

- Establishes an antibiotic resistance monitoring program due to the use of antibiotics in animal production (terrestrial and aquatics).
- Establishes the responsibilities, obligations, quality levels and control of reference laboratories and others that carry out AMR surveillance.

- **Factor: Governance**

Institutional governance under the One Health approach

282. The country has established a National Coordination Committee on AMR (hereafter the Committee). If your answer is No, indicate the main reasons.
283. The Committee has been formalized.
284. The Committee has been constituted under an intersectoral approach, that is to say it considers in a balanced way the areas of public health, food safety, the environment, production and animal health (terrestrial and aquatic).
285. Indicate the official date the Committee was formed. Indicate the composition of the National Intersectoral Coordination Committee on AMR. Indicate the public institutions that are represented in the Committee, for example, public health, animal health (terrestrial and aquatic), plant health, food safety and the environment, among others; together with the positions of the representatives of the institutions.
286. Indicate other sectors that are part of the Committee (food industry, food industry for animal use, pharmaceutical industry, academy, organizations of agricultural producers, international organizations, others).
287. The Committee, where appropriate, extends invitations to other professionals, for example: International organizations, academics, professional associations, others.
288. Indicate the institution that coordinates or chairs the Committee. Indicate name and title of the president or coordinator.
289. Indicate the frequency of Committee meetings.
290. Indicate whether the Committee has supra-ministerial political support, for example, through government support.
291. Indicate whether the Committee has legal support (norms, resolutions, others) for its statutes, intersectoral composition and operation.
292. Indicate whether the norm or legal institution of the Committee gives explicit responsibilities to the agriculture sector.
293. Indicate whether the Committee has specific funds for the management of its activities. Indicate whether these are national and/or international.
294. Indicate whether the Committee has a support secretariat. If your answer is yes, please provide contact information.
295. Indicate whether the Committee has formed a technical support group.
296. Indicate whether the Committee has established communication and coordination channels between the sectors and member institutions in a fluid and permanent manner.
297. Indicate whether the country has developed a national analysis and evaluation of the AMR situation prior to the elaboration of the National Intersectoral Action Plan on antibiotic resistance.
298. The country has a National Action Plan on AMR (hereafter, National Action Plan).
299. Indicate whether the National Action Plan has been formulated by the Committee (or by the technical group designated by the Committee).
300. Attach the document of the National Action Plan.
301. The National Action Plan has legal support to formalize its implementation.
302. The National Action Plan is based on a national strategy that proportionally and comprehensively represents the key sectors for the containment of the AMR indicated below:
    - Public health

- Food safety
- Environment
- Production and health of terrestrial animals
- Production and health of aquatic animals
- Agriculture

303. The AMR activities outlined in the National Action Plan are incorporated into the operational plans of the respective institutions involved in its implementation.
    - Livestock sector
    - Aquaculture sector
    - Human health
304. Indicate whether the National Action Plan addresses the 5 strategic objectives of the Global Action Plan on AMR of the World Health Organization.
305. Indicate whether the National Action Plan has a specific budget to carry out its activities, whether this is overall or by institution.

Governance in the agri-food sector

306. The high administration of the Livestock, Aquaculture and Agricultural Health Services and Food Safety Services, consider AMR a priority of the institution:
    - Animal health services;
    - Aquaculture health services;
    - Agricultural health services;
    - Food safety services.
307. Also, assess whether AMR is a priority for the department directors of Livestock, Aquaculture and Agricultural Health Services and Food Safety Services:
    - Animal health services;
    - Aquaculture health services;
    - Agricultural health services;
    - Food safety services.
308. Indicate the departments of Livestock, Aquaculture and Agricultural health Services and Food Safety Services that are addressing the issue of AMR, together with the names and titles of a representative in each department.
309. Indicate the name of the person in charge who acts as coordinator or representative of the AMR activities within the Livestock, Aquaculture and Agricultural Health Services and Food Safety Services.
310. Indicate the department(s) that acts as coordinator and representative of the AMR activities of the Livestock, Aquaculture and Agricultural Health Services and Food Safety Services at the National Committee level.
311. Indicate the main programs/strategies in operation that contribute to the containment of AMR in the agri-food sector (including aquaculture).

Supranational programs

312. The country participates in supranational initiatives (for example, international organizations, regional committees or networks, etc.) for the containment of AMR. If the answer is yes, indicate whether these initiatives are in line with the National Action Plan.

- **Factor: Communication, Awareness and training**

Veterinary Medical Competencies of the Official Service (terrestrial and aquaculture)

313. For the topics listed below, estimate what percentage of veterinarians from the Official Veterinary Service have knowledge about:

- adequate use of antibiotics and maintenance of antibiotic use records;
- concept of withdrawal period when using antibiotics in production animals;

- the withdrawal period as a method to prevent the appearance of residues in animal products for human consumption;
- place to obtain updated and reliable information regarding the specific withdrawal periods for the different products;
- the main mechanisms that produce antibiotic resistance in bacterial agents;
- place to obtain information regarding the link of the use of antibiotics in production animals for human consumption and the development of resistance pathogens of importance in human medicine;
- use and elimination of antibiotics caring the safety of the food chain and the safety of the environment;
- international or regional tools or methodologies that allow mitigating the development of antibiotic resistance in animal production;
- promotion of the good antibiotic use in animal production.

Raising awareness, technical training and sanitary education

314. From the communication units of the Service:

a. Indicate if in the last two years, whether significant information and dissemination activities have been carried out on the risks of AMR and its mitigation measures. If your answer is yes:

- Indicate the audiences or stakeholders: medium and large producers; family production; food industry; veterinarians from the private sector; agricultural technicians from the private area; civil society.
- Indicate the main communication instruments used for these purposes.

b. Indicate if a representative of the communications unit of the Official Service or of the Ministry of Agriculture participates in the meetings of the AMR Inter-sectorial Committee, when appropriate.

c. Indicate the level of development of the communications strategy or campaign to meet Objective 1 of the National Action Plan.

d. Assess the level of relationship between the communications unit of the Official Service and the technical units linked to AMR.

e. Indicate if AMR is part of the operational program of the communications unit of the Official Service.

f. Determine the level of relationship between the communications unit of the Service and the communication units of the Ministry of Health.

g. Determine the level of relationship with the country's mass media.

h. Determine if the Official Service has trained spokespersons to address the issue of AMR within the mass media and civil society.

315. Indicate if the technical units (departments) of the Official Service linked to AMR have carried out technical training sessions for professionals and technicians in matters related to AMR in the last two years.

316. If your previous answer is Yes, indicate the groups that have benefitted from these training sessions and their main topics:

- professionals and technicians of the official service (including accredited);
- professionals and technicians from the private sector;
- other actors in the food chain (identify which ones).

317. Indicate whether the technical units (departments) of the Official Service linked to AMR have carried out health education campaigns (rural training) to small livestock and aquaculture producers (family farming) in the last two years:

- livestock producers;
- aquaculture producers.

318. If your answer is yes, indicate the type of educational methodology used for rural training:

- methodology of livestock producers;
- methodology of aquaculture producers.

Indicate whether rural training is carried out with the support of the following entities:

- public health;
- state entity for productive development;
- NGOs;
- international organizations;
- universities;
- rural extension services;
- municipalities or local governments;
- other (indicate).

319. Indicate the main topics addressed in rural training campaigns (for example: AMR risks, good sanitary practices, good antimicrobial use practices, good environmental practices, other) (small livestock and aquaculture producers). Assess the level of capabilities of the Official Service to design and deploy health education strategies (rural training) in good, regular and bad.

320. For small livestock and aquaculture producers, estimate the percentage of coverage of the rural area that has been the recipient of rural training campaigns.

- **Factor: Research and innovation**

Research and innovation

321. Identify institutions or work groups that are studying antibiotic resistance, the development of new antibiotics and alternatives to antibiotic use.

National experts in antibiotic resistance

322. In the table below, indicate the antibiotic resistance experts available in your country.

3 Annex 3. Example of the application of the methodology.

The estimation of the subfactor final risk is made by crossing the result obtained from the probability of occurrence of each subfactor of risk and the general effectiveness of the associated measure (s). Below is an example, step by step (1 to 3), on how the methodology is applied (Table 2, Table 7, Table 9). This example does not reflect any country in particular.

Step 1: Estimation of the probability of risk occurrence

Based on the responses provided in the survey and a standard established by the FAO team, each risk subfactors of introduction and exposure is categorized into three levels of probability of occurrence: high probability of occurrence, moderate probability of occurrence and low probability of occurrence (Table 1).

Table 1. Criteria to estimate risk occurrence probability

Probability of risk occurrence	Definition
**High**	Given the characteristics of the hazard and the situation analysed, the event occurs frequently (always or almost always).
**Moderate**	Given the characteristics of the hazard and the situation analysed, the event occurs or may occur on a regular basis (on some occasions).
**Low**	Given the characteristics of the hazard and the situation analysed, the event is rare, but does occur.

Those risk subfactors for which no response is provided or a lack of information is indicated are classified as having a high probability of risk. In the event that the subfactor does not apply to the production system, it is evaluated with a low probability of risk.

Table 2 shows an example of the estimation of the probability of occurrence of the subfactors sanitary situation, veterinary assistance and dependence on the use of antibiotics.

The cut-off points (for estimation of the probability of occurrence and effectiveness of mitigation measures) where validated during five expert elicitation process conformed by multiple professionals from the public and private sector with expertise in animal production (terrestrial, aquatic), feed, family farming, public health and epidemiology. This standard can be modified according to expert studies and other assumptions.

Table 2. Categorization of the probability of occurrence of the subfactors sanitary situation, veterinary medical assistance and dependence on the use of antibiotics. Animal production system: Broiler chickens.

Risk subfactor	Standard		Country answer	Probability of occurrence
Sanitary situation (risk for human health)	Bacteria declared on the OIE list[1], with scientific evidence of AMR in animal production	High	Avian mycoplasmosis (*Mycoplasma gallisepticum*)	High
	Bacteria declared on the OIE list[1], with scientific evidence of AMR in animal production, or	Moderate		
	Bacteria not declared on the OIE list[1], without scientific evidence of AMR in animal production	Low		
Veterinary assistance or other professional recognized by the competent authority	Less than or equal to 60%	High	80	Low
	Between 61% and 79%	Moderate		
	Greater than or equal to 80%	Low		
Dependence of the production system in the use of antibiotics	Greater than or equal to 60%	High	65	High
	Between 31% and 59%	Moderate		
	Lower or equal to 30%	Low		

[1] Chapter 1.3 Sanitary Code for terrestrial animals. https://www.oie.int/index.php?id=169&L=2&htmfile=chapitre_diagnostic_tests.htm/ Chapter 1.3 Sanitary Code for aquatic animals.https://www.oie.int/index.php?id=171&L=2&htmfile=chapitre_diseases_listed.htm

Step 2: Estimation of effectiveness of mitigation measures

According to the answers given in the survey, the measures are evaluated regarding their effectiveness and characterized as high, moderate and low (Table 3).

Table 3. Criteria to estimate effectiveness of mitigation measures

Effectiveness of mitigation measures	Definition
**High**	Mitigation measures are effective in preventing or controlling the hazard.
**Moderate**	Mitigation measures are moderately effective in preventing or controlling the hazard.
**Low**	Mitigation measures are not very effective in preventing or controlling the hazard.

Mitigation measures are evaluated at the individual and general levels. The latter is applied only in the case of having more than one measure to contain the same risk subfactor.

a. Individual effectiveness

In the case of programs and vaccines, their existence is evaluated as a high level of effectiveness, while the lack of such programs or vaccines is evaluated as low effectiveness. Legislation is evaluated according to its existence[2], quality[3], coverage[4] and quality of application[5], according to the standards shown in Table 4. If there is no existing legislation, the attributes of quality, coverage and quality of application are not evaluated, thereby obtaining a low level of effectiveness.

Table 4. Categorization of individual effectiveness of legislation

Evaluation indicator	Effectiveness		
	High	Moderate	Low
**Existence**	Yes	Yes	Yes /No
**Quality**	Yes	Yes	Yes /No
**Coverage**	National	National/Specific	National/Specific
**Quality of application**	≥ 80%	≥ 60%[6]	< 60%

If no answer was provided for the mitigation measure, or it was indicated that there is no information available, it was classified as having a low level of effectiveness.

Those risk mitigation measures for which no response is provided or a lack of information is indicated are classified as having a low level of effectiveness.

b. General effectiveness

After the individual assessment, a general effectiveness of the mitigation measures is carried out. Each mitigation measure evaluated is given a score depending on the level of individual effectiveness (Table 5). For those risk

[2] Indicates whether or not regulation exists regarding the issue consulted.
[3] Indicates whether the legislation is designed to fulfill the stated objective.
[4] Indicates whether the legislation is applied at the national level or to a specific group.
[5] Indicates whether the legislation is applied correctly.
[6] In order for a mitigation measure with national coverage to be classified as moderately effective, it must have a quality of application of less than 79%.

subfactors with more than one mitigation measure, an average is calculated from the individual scores of the corresponding mitigation measures, which represents the general level of effectiveness (

Table 6). In those subfactors with only one mitigation measure, their general effectiveness is the same as their individual effectiveness.

Table 5. Numerical value according to level of individual effectiveness categorization

Individual effectiveness	Score
**High**	3
**Moderate**	2
**Low**	1

Table 6. Categorization of general effectiveness according to average of individual numerical values

Average of individual scores	General effectiveness
**1.0 a 1.5**	Low
**1.6 a 2.5**	Moderate
**2.6 a 3**	High

Table 7 shows an example of the estimation of the effectiveness of the mitigation measures for the subfactors sanitary situation, veterinary assistance and dependence on the use of antibiotics.

Table 7. Evaluation of the effectiveness of the mitigation measures for the subfactors sanitary situation, veterinary medical assistance and dependence on the use of antibiotics. Risk to animal health from terrestrial animal production.

Risk subfactor	Mitigation measure	Country response							
		Existence	Quality	Coverage	Quality of application	Individual effectiveness		Total effectiveness	
						Level	Score	Level	Score
**Sanitary situation (*Avian mycoplasmosis* (Mycoplasma gallisepticum)**	Application of public and/or private programs for the implementation of good animal production practices	No answer	-	-	-	Low	1	1.8	Moderate
	Existence of vaccines	Yes	-	-	-	High	3		
	Legislation on animal production that establishes a surveillance system framework (collection, transmission and use of epidemiological data) for endemic bacterial diseases of importance for the country	No*	-	-	-	Low	1		
	Legislation that establishes sanitary measures for the prevention of diseases in production animals such as: good production practices and biosecurity	Yes	Yes	National	60	Moderate	2		
	Legislation that establishes sanitary measures for the control of diseases in production animals, such as good production practices and biosecurity	Yes	Yes	National	60	Moderate	2		
**Veterinary assistance or other professional recognized by the competent authority**	Application of public and/or private programs for the implementation of good animal production practices	Yes	-	-	-	High	3	3	High
**Dependence on the use of antibiotics**	Application of public and/or private programs for the implementation of good animal production practices	Yes	-	-	-	High	3	2.3	Moderate
	Level of existing knowledge regarding the use of alternatives to antibiotics	Yes	Yes	Yes	85	High	3		
	Legislation that establishes the requirements for the use of antibiotics in animal production	No*	-	-	-	Low	1		

*In the absence of legislation, the effectiveness aspects (quality, coverage and quality of application) are not evaluated, and therefore a low individual effectiveness level is achieved.

Step 3: Estimation of final risks by risk subfactor

The estimation of the final risk by subfactor is made by crossing the result obtained from the probability of occurrence of each subfactor of risk and the general effectiveness of the associated measure (s) (Table 8).

Table 8. Risk estimation matrix

Probability of occurrence	Effectiveness of mitigation measures		
	**High**	**Moderate**	**Low**
**High**	Low	Moderate	High
**Moderate**	Low	Moderate	Moderate
**Low**	Low	Low	Low

It is important to inspect not only the final risk but also the results of probability of occurrence and effectiveness of the mitigation measure (s) associated with each risk subfactor independently. As shown in Table 8**Table 8**, when faced with high levels of effectiveness of mitigation measures, low final risks are obtained, even when faced with high or moderate probabilities of risk. The risks are never zero and therefore must always be considered. Additionally, the identification of gaps regarding the mitigation measures for certain risks serves as the basis for improvements regarding the containment of AMR.

Table 9. Estimation of final risks according to the risk subfactors sanitary situation, veterinary assistance and dependence on the use of antibiotics. Risk to animal health from terrestrial animal production.

Risk subfactor	Probability of occurrence	Effectiveness of the measure(s)	Final risk
**Sanitary situation (risk to human health)**	High	Moderate	Moderate
**Veterinary assistance or other professional recognized by the competent authority**	Low	High	Low
**Dependence on the use of antibiotics**	High	Moderate	Moderate

4 Annex 4. Comparative results

Figure 1. Percentages of subfactors that present gaps based on the qualitative risk assessment for animal health, by risk factor and animal production system.

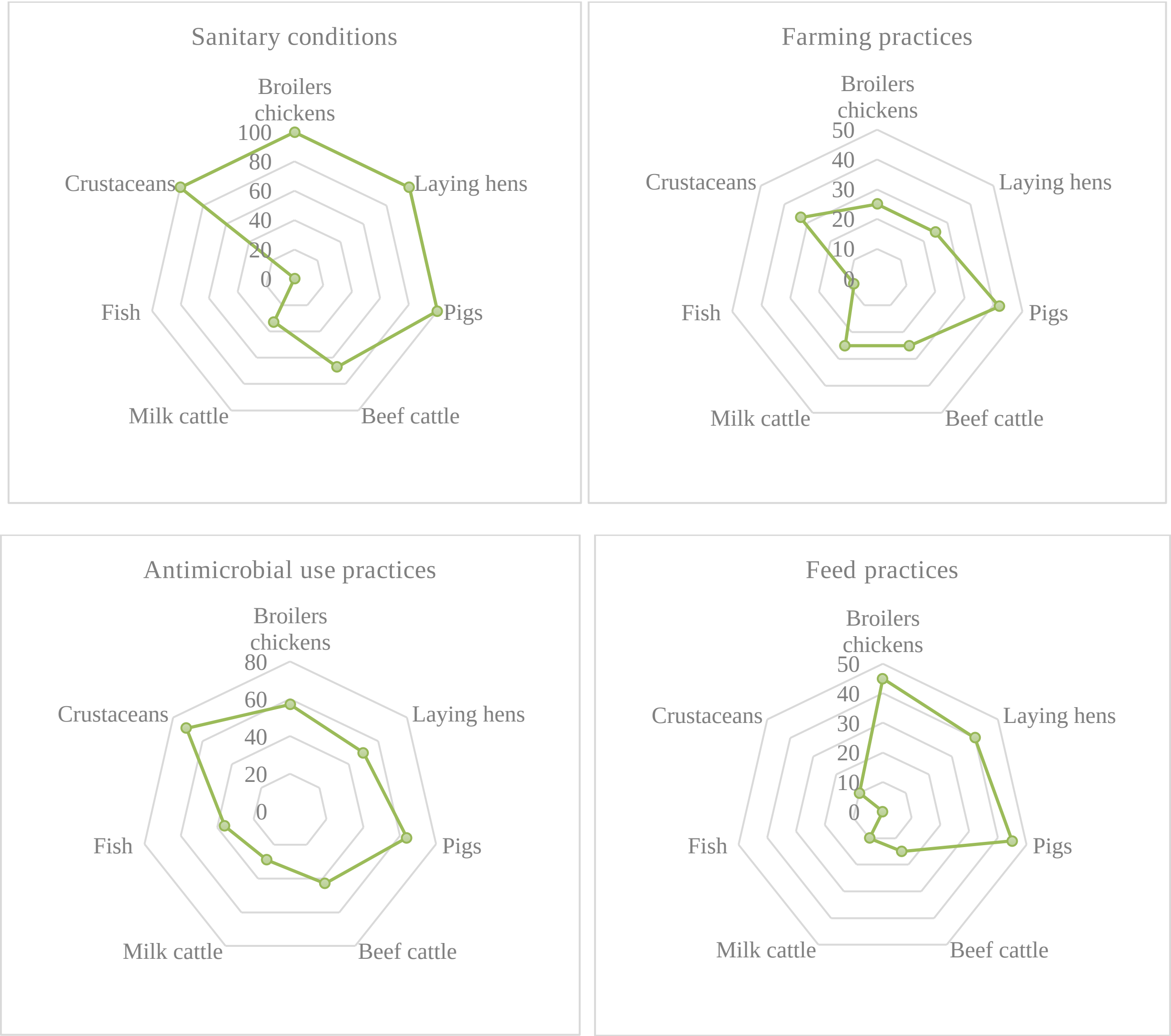

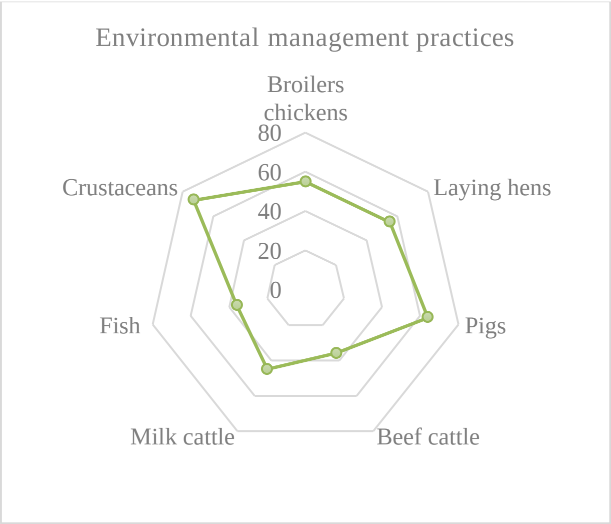

Figure 2. Percentages of subfactors that present gaps based on the qualitative risk assessment for human health, by risk factor.

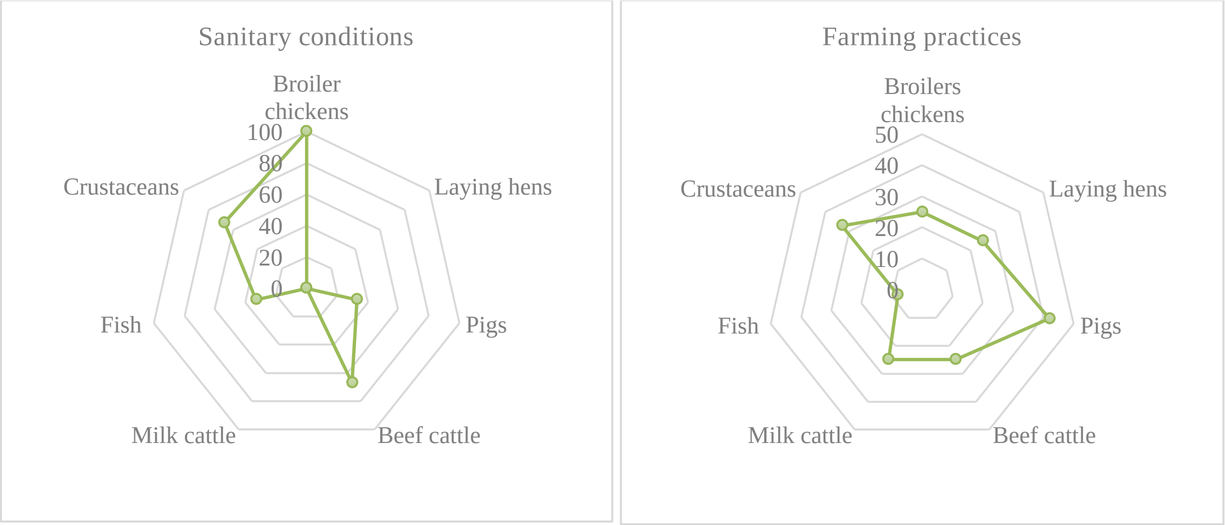

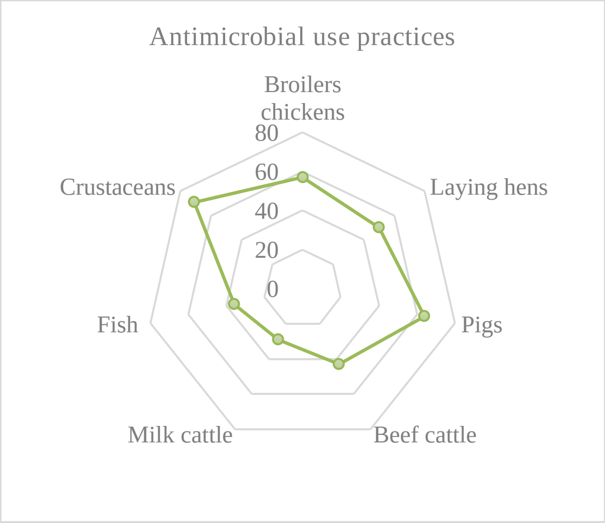
Antimicrobial use practices
Broilers chickens
Laying hens
Pigs
Beef cattle
Milk cattle
Fish
Crustaceans
80
60
40
20
0

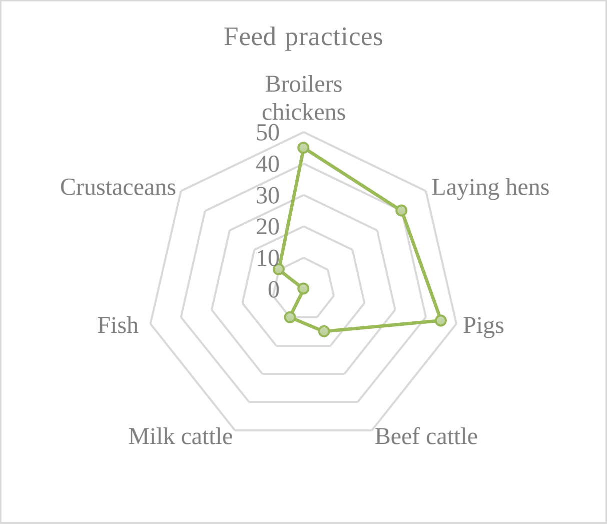
Feed practices
Broilers chickens
Laying hens
Pigs
Beef cattle
Milk cattle
Fish
Crustaceans
50
40
30
20
10
0

,

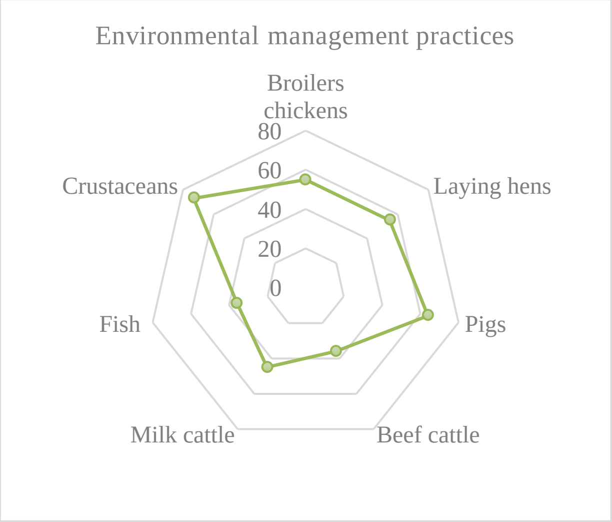
Environmental management practices
Broilers chickens
Laying hens
Pigs
Beef cattle
Milk cattle
Fish
Crustaceans
80
60
40
20
0

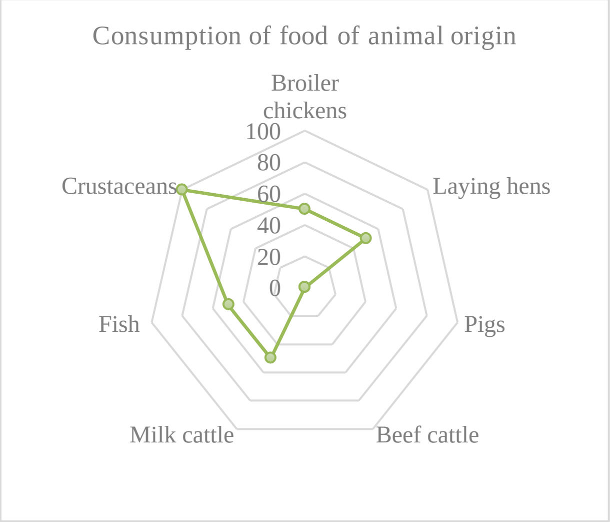
Consumption of food of animal origin
Broiler chickens
Laying hens
Pigs
Beef cattle
Milk cattle
Fish
Crustaceans
100
80
60
40
20
0

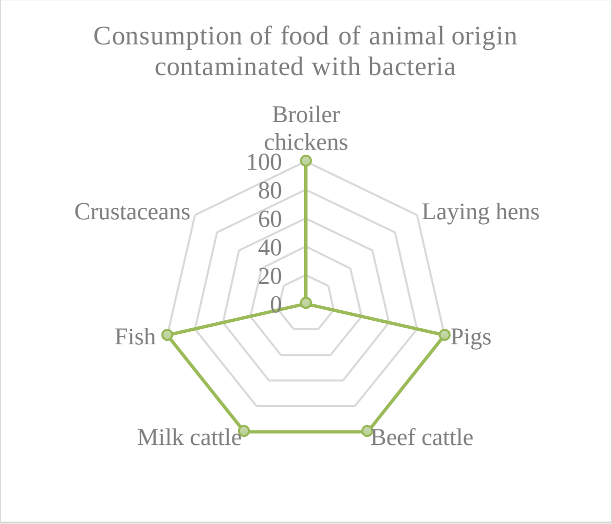
Consumption of food of animal origin contaminated with bacteria
Broiler chickens
Laying hens
Pigs
Beef cattle
Milk cattle
Fish
Crustaceans
100
80
60
40
20
0

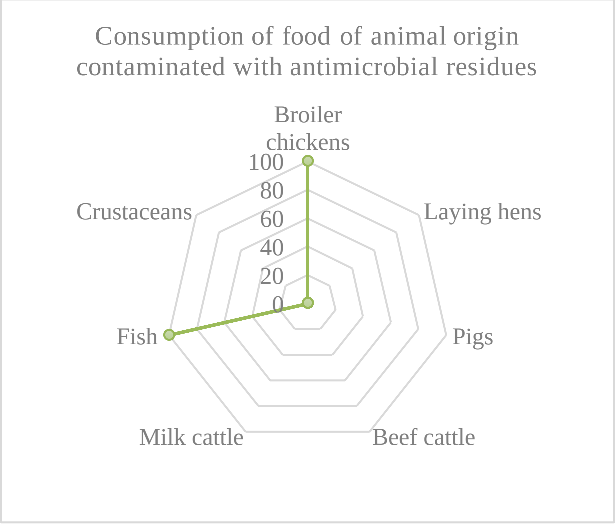
Consumption of food of animal origin contaminated with antimicrobial residues
Broiler chickens
Laying hens
Pigs
Beef cattle
Milk cattle
Fish
Crustaceans
100
80
60
40
20
0

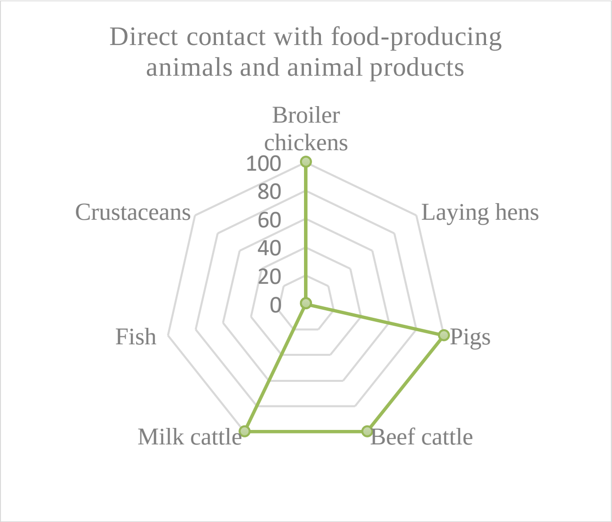
Direct contact with food-producing animals and animal products
Broiler chickens
Laying hens
Pigs
Beef cattle
Milk cattle
Fish
Crustaceans
100
80
60
40
20
0
